# Fibroblast activation protein targeted radiotherapy induces an immunogenic tumor microenvironment and enhances the efficacy of PD-1 immune checkpoint inhibition

**DOI:** 10.1007/s00259-023-06211-6

**Published:** 2023-04-22

**Authors:** Dirk Zboralski, Frank Osterkamp, Esben Christensen, Anne Bredenbeck, Anne Schumann, Aileen Hoehne, Eberhard Schneider, Matthias Paschke, Jan Ungewiss, Christian Haase, Liliane Robillard, Andrew D. Simmons, Thomas C. Harding, Minh Nguyen

**Affiliations:** 1 3B Pharmaceuticals GmbH, Magnusstraße 11, D-12489 Berlin, Germany; 2 Minerva Imaging ApS, Ølstykke, Denmark; 3 Clovis Oncology, Inc., CO Boulder, USA

**Keywords:** FAP, PD-1, TRT, Theranostic, STING, CD8

## Abstract

**Purpose:**

FAP is a membrane-bound protease under investigation as a pan-cancer target, given its high levels in tumors but limited expression in normal tissues. FAP-2286 is a radiopharmaceutical in clinical development for solid tumors that consists of two functional elements: a FAP-targeting peptide and a chelator used to attach radioisotopes. Preclinically, we evaluated the immune modulation and anti-tumor efficacy of FAP-2287, a murine surrogate for FAP-2286, conjugated to the radionuclide lutetium-177 (^177^Lu) as a monotherapy and in combination with a PD-1 targeting antibody.

**Methods:**

C57BL/6 mice bearing MCA205 mouse FAP-expressing tumors (MCA205-mFAP) were treated with ^177^Lu-FAP-2287, anti-PD-1, or both. Tumor uptake of ^177^Lu- FAP-2287 was assessed by SPECT/CT scanning, while therapeutic efficacy was measured by tumor volume and survival. Immune profiling of tumor infiltrates was evaluated through flow cytometry, RNA expression, and immunohistochemistry analyses.

**Results:**

^177^Lu-FAP-2287 rapidly accumulated in MCA205-mFAP tumors leading to significant tumor growth inhibition (TGI) and longer survival time. Significant TGI was also observed from anti-PD-1 and the combination. In flow cytometry analysis of tumors, ^177^Lu-FAP-2287 increased CD8^+^ T cell infiltration which was maintained in the combination with anti-PD-1. The increase in CD8^+^ T cells was accompanied by an induction of STING-mediated type I interferon response and higher levels of co-stimulatory molecules such as CD86.

**Conclusion:**

In a preclinical model, FAP-targeted radiotherapy enhanced anti-PD-1-mediated TGI by modulating the TME and increasing the recruitment of tumor-infiltrating CD8^+^ T cells. These findings provide a rationale for clinical studies of combined ^177^Lu-FAP-2286 radiotherapy and immune checkpoint inhibition in FAP-positive tumors.

**Supplementary Information:**

The online version contains supplementary material available at 10.1007/s00259-023-06211-6.

## Introduction

Immunotherapy utilizing immune checkpoint inhibitors (ICIs) such as antibodies targeted to programmed cell death 1 (PD-1) have been established as an essential treatment against cancer [1]. However, tumor intrinsic and acquired resistance to ICIs limits their clinical utility [2, 3]. The resistance to ICIs is in part mediated by immune-suppressive cells in the tumor microenvironment (TME), such as fibroblast activation protein (FAP)-expressing cancer-associated fibroblasts (CAFs) [4]. In addition to its roles in tumor proliferation, angiogenesis, and metastasis that are crucial for cancer evolution and progression, CAFs can exert immunosuppressive influences on the immune populations in the TME [5]. By regulating immune cell infiltration and phenotype within the TME, CAFs influence their spatial localization and functionality. For instance, CAFs are known to increase the attraction, survival, and activation of regulatory T cells (Tregs). CAF subsets with distinct extracellular matrix (ECM) programs are associated with T cell exclusion from the tumor and correlate with PD-1^+^ and CTLA4^+^ CD4^+^ T cell content in tumor lesions [6, 7]. Furthermore, CAFs and macrophages contribute to ECM remodeling to form a desmoplastic TME that prevents T cells infiltrating to the core of the tumor [8]. CAFs also secrete factors into the circulation that bind to and differentiate macrophages which in turn regulate CAFs via TGFB1, thus promoting the secretion of matrix metalloproteinases and collagen which contributes to ECM remodeling [8]. A better understanding of the roles of CAFs will help identify combinations that can be used to establish an immunogenic TME and enhance anti-tumor responses.

Targeted radionuclide therapy (TRT) has emerged as a new class of anti-cancer agents [9, 10]. It consists of a tumor targeting moiety linked to a radionuclide that allows the selective delivery of radiation to cancer cells. In addition to direct cytotoxic effects, radiation-based therapeutics can promote inflammatory signals and increase tumor immunogenicity. Evidence from preclinical and clinical studies suggest that TRT can promote an immune-privileged TME [11]. In a previous report with beta-particle emitting radionuclides, radiation induced DNA damage was demonstrated to upregulate pro-inflammatory cytokines such as interferons through activation of the stimulator of interferon genes (STING) pathway [12]. These cytokines can recruit immunostimulatory immune cells into the TME, including T cells, antigen presenting cells, and natural killer cells [13, 14] and impede immunosuppressive immune cells, including Tregs [15].

The radiopharmaceutical FAP-2286 was developed for the imaging and treatment of FAP-expressing cancers. This compound is comprised of a cyclic FAP-binding peptide linked to tetraazacyclododecane tetraacetic acid (DOTA), allowing radionuclide chelation for imaging or therapeutic applications. A preclinical study demonstrated rapid accumulation and long retention of lutetium-177 (^177^Lu)-radiolabeled FAP-2286 in FAP-positive tumors resulting in significant tumor growth suppression, with minimal uptake in normal organs [16]. In the first-in-human experience of 11 patients, FAP-2286 has established its utility as a theranostic agent labeled with gallium-68 (^68^Ga) for positron emission tomography (PET) imaging and with ^177^Lu for treatment of patients. PET scans of patients administered ^68^Ga-FAP-2286 confirmed significant uptake in neoplastic lesions coupled with low uptake in normal tissues of patients with either pancreatic, breast, ovarian, or colorectal carcinomas. As a therapeutic radiopharmaceutical, ^177^Lu-FAP-2286 showed similar tumor specific biodistribution by single-photon emission computed tomography (SPECT) imaging as ^68^Ga-FAP-2286, coupled with a long tumor retention time that appeared to improve symptoms manifested by pain reduction in 3 patients with advanced disease [17].

Herein, we demonstrate that combined therapies using radiolabeled FAP-targeting agent ^177^Lu-FAP-2287, a murine surrogate of ^177^Lu-FAP-2286, together with PD-1 checkpoint blockade induce tumor inhibition in a FAP expressing MCA205 fibrosarcoma tumor model. The efficacy of this combination is mediated by radiation-induced modulation towards an immunogenic TME. Our findings support the clinical investigation of ^177^Lu-FAP-2286 in combination with anti-PD-1 blockade.

## Materials and Methods

Detailed information on all materials and methods including peptide synthesis are available in the Supplementary Materials and Methods.

### Cell lines and reagents

Stably expressing mouse FAP clone MCA205-mFAP was generated by lentiviral vector (System Biosciences) transduction of MCA205 mouse fibrosarcoma cells (Sigma) and subsequent puromycin selection, in the manufacturer’s recommended medium. Puromycin resistance cells were then cloned by single cell dilution and FAP expression was screened by flow cytometry (Accuri C6, BD Biosciences). Cells were free of mycoplasma contamination.

### Immunohistochemistry (IHC)

Tumors were formalin-fixed and paraffin-embedded and processed into 4 μm tissue sections. Tumor sections were stained for FAP (EPR20021, Abcam), CD8 (D4W2Z, Cell Signaling), and CD4 (EPR19514, Abcam) on a Bond Rx autostainer (Leica Biosystems) with Bond Polymer Refine Detection (Leica Biosystems) according to the manufacturer's protocol. After staining, sections were dehydrated and film cover slipped using a TissueTek-Prisma and Coverslipper (Sakura). Whole slide scanning (40x) was performed on an Aperio AT2 (Leica Biosystems). Image analysis and quantification was performed on Aperio ImageScope v12.1 (Leica Biosystems) or QuPath v0.1.2 [18].

### Autoradiography (ARG)

In vitro FAP receptor ARG was performed as previously described with minor modifications [19]. Frozen tissue Sects. (20 μm) were incubated with ^111^In-labeled FAP-2287 (15 MBq/nmol). Subsequently, an x-ray film (Biomax MR films; Carestream Health) was placed onto positioned slides and exposed for 2 days. Relative optical film density was determined using MCID™ Core 7.0 Rev 2.0 software (InterFocus) and correlated with known amounts of radioactivity via a separately recorded calibration curve.

### Tumor in vivo biodistribution study

All animal experiments were carried out under licenses approved by the respective authorities and in compliance with guidelines on animal welfare. Female C57BL/6 mice (n = 6) were subcutaneously implanted with 1 × 10^6^ MCA205-mFAP cells. For single-photon emission computed tomography (SPECT) imaging, 30 MBq (1 nmol) ^177^Lu-FAP-2287 was administered by intravenous injection (100 µL) when mean tumor volume (MTV) was 126 mm^3^ (mean body weight 20.4 g) and the radiotracer distribution was assessed at 3, 24, 48 and 72 h post injection (pi) using the nanoScan SPECT/CT system (Mediso). Animals were anesthetized (isoflurane 3% in 100% O_2_) and eye lubricants were applied before each scan. Study was performed at Minerva Imaging ApS (Denmark).

### Tumor in vivo efficacy and pharmacodynamics studies

Female C57BL/6 mice were subcutaneously implanted with 1 × 10^6^ MCA205-mFAP cells (n = 12 per group). For tumor efficacy evaluation, a single dose of 60 MBq (1 nmol) ^177^Lu-FAP-2287 was administered by intravenous injection (100 µL), and 10 mg/kg anti-PD-1 (RMP1-14, Bio X Cell) was given by intraperitoneal injection (10 mL/kg) twice weekly for 6 doses starting the following day. Treatment began when the MTV was 94 mm^3^ (mean body weight 20.4 g). For pharmacodynamic evaluation, female C57BL/6 mice (n = 8 per group) were subcutaneously implanted with 1 × 10^6^ MCA205-mFAP cells and treated with 60 MBq (1 nmol) ^177^Lu-FAP-2287 as a single dose (100 µL), and 10 mg/kg anti-PD-1 twice weekly (10 mL/kg) starting the following day for 3–4 doses. Treatment began when MTV was 162 mm^3^ (mean body weight 20.8 g) and tumors were collected at days 8 and 13 pi for analysis.

### Ex vivo immune profiling by flow cytometry

Tumors were dissected into small pieces, digested using a tumor dissociation enzyme mix (Miltenyi Biotec) at 37 °C for 40 min in a shaking water bath, processed through 70 μM cell strainers and counted (NucleoCounter NC-202, Chemotec). Cells (10 × 10^6^) were Fc-blocked (2.4G2, BD Biosciences) and stained in FACS buffer (PBS with 0.5% BSA, 0.1% NaN_3_ and 2 mM EDTA) with Brilliant Stain Buffer Plus (BD Biosciences) for surface markers CD45 (30-F11), CD3 (145-2C11), CD4 (RM4-5), CD8 (53–6.7), CD25 (PC61), CD127 (SB/199), Ly6g (1A8), Ly6c (HK1.4), CD11b (M1/70), CD86 (GL1), CD314 (CX5), F4/80 (T45-2342), and eFlour 780 fixable viability dye (Thermo Fisher Scientific) for 30 min on ice. Anti-Ly6c was purchased from Biolegend, while remaining antibodies were from BD Biosciences. Samples were washed and acquired on the FACSymphony A3 and analyzed in FlowJo v10.8.1 (BD Biosciences, Supplementary Fig. S1). The efficacy and pharmacodynamics studies were performed at Minerva Imaging ApS, (Denmark).

### RNA expression analysis

Total RNA was isolated from tumors using the PureLink RNA Mini kit (Thermo Fisher Scientific) following the manufacturer’s protocol. The nCounter PanCancer Immune Profiling (mouse) panel (NanoString Technologies) was used to measure the expression in the RNA samples. Following hybridization, transcripts were quantitated and analyzed using the nCounter Digital Analyzer and nCounter Advanced Analysis Software v4.0 (NanoString Technologies). Data were normalized for assay efficiency by multiplying each count by a positive normalization factor obtained for each sample. For differential expression analysis, the Benjamini-Yekutieli statistical analysis, which calculates false discovery rate, was employed to generate an adjusted p-value.

## Results

### Biochemical and cellular characterization FAP-2287

FAP-2286 and its metal complexes demonstrate potent and selective binding to human FAP [16], however, a significant reduction in affinity was observed for mouse FAP despite the high homology of the proteins (89%) [20]. As such, for preclinical studies targeting mouse FAP, a closely related surrogate of FAP-2286, termed FAP-2287 [21] was evaluated as a substitute (Supplementary Fig. S2A). FAP-2287 was identified in a peptide screen for mouse FAP affinity and found to have the similar affinity towards murine FAP as FAP-2286 to human FAP when both are chelated with ^177^Lu. The affinity and selectivity of FAP-2287 along with its complex of natural nonradioactive lutetium (^nat^Lu-FAP-2287) were evaluated in vitro.

Surface plasmon resonance (SPR) analysis of FAP-2287 and ^nat^Lu-FAP-2287 demonstrated potent binding to human FAP with mean equilibrium dissociation constant (K_D_) values of 0.4 and 0.1 nM, respectively, and to mouse FAP with 1.2 and 0.5 nM, respectively (Supplementary Fig. S2B). To further characterize binding in vitro, the potency of the compounds was measured against FAP endopeptidase enzymatic activity on a fluorophore-labeled substrate. FAP-2287 and ^nat^Lu-FAP-2287 inhibited FAP enzymatic activity with mean IC_50_ values of 1.4 and 1.3 nM and 5.1 and 3.3 nM against human and mouse FAP, respectively (Supplementary Fig. S2B). The binding of FAP-2287 to cell surface FAP was evaluated in a competition assay against a fluorophore-labeled competitor peptide using the human WI-38 fibroblast-like fetal lung cell line that endogenously expresses FAP. FAP-2287 and ^177^Lu-FAP-2287 binding to FAP markedly reduced fluorophore-labeled competitor peptide bound to cells with a mean IC_50_ of 3.9 and 2.3 nM, respectively, as measured by flow cytometry (Supplementary Fig. S2B).

Taken together, the affinity of ^nat^Lu-FAP-2287 towards murine FAP is similar compared with the affinity of clinical-stage ^nat^Lu-FAP-2286 towards human FAP [16], making FAP-2287 an appropriate murine substitute. Furthermore, selectivity for binding to related family members and plasma stability were comparable to previous results for FAP-2286 (Supplementary Fig. S2B; [16]).

### In vivo biodistribution of ^177^Lu-FAP-2287 by SPECT/CT imaging

**Fig. 1:**
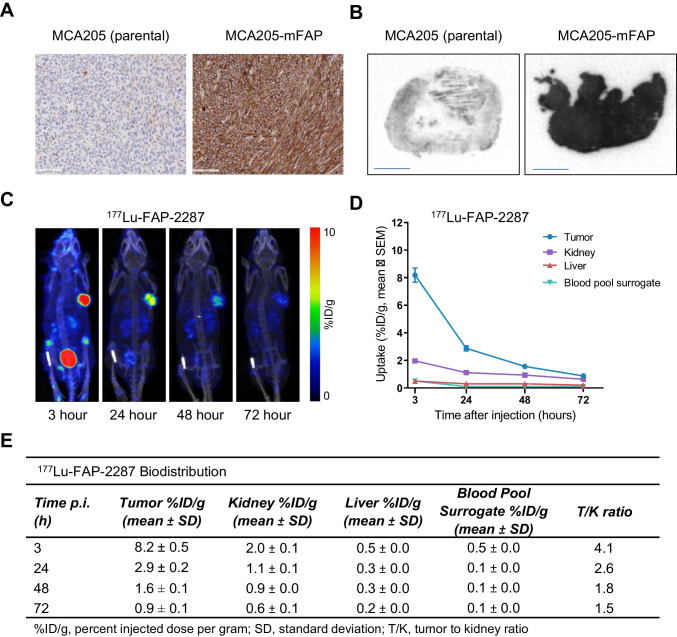
In vivo uptake of ^177^Lu-FAP-2287 into FAP-positive MCA205-mFAP syngeneic tumors. Representative images of MCA205-mFAP tumors analyzed by FAP IHC (**A**, 200 μm) and ^111^In-FAP-2287 ARG (**B**, 2 mm) are shown. SPECT images of one representative mouse at 4 different timepoints are shown (**C**). Quantification of ^177^Lu-FAP-2287 biodistribution as mean ± SEM %ID/g (n = 6) in tumor and kidney at 3, 24, 48 and 72 h pi (**D**) and summary table of uptake in several organs and tumor to kidney ratios (**E**) are shown

The MCA205 fibrosarcoma cell line was engineered to express murine FAP, after initial screening of multiple syngeneic and GEMM tumors showed minimal to no FAP staining (Supplementary Fig. S3) in contrast to patient tumors that are commonly infiltrated with significant FAP-positive CAFs [16]. Prior to in vivo evaluation of FAP-2287, tumors derived from MCA205-mFAP cell implantation in C57BL/6 mice were assessed for murine FAP expression by IHC and FAP-2287 binding by in vitro receptor ARG. In comparison to the low level of FAP expression observed in MCA205 tumors, MCA205-mFAP tumors displayed high, homogeneous FAP levels by IHC, which was corroborated with a strong ^111^In-FAP-2287 binding signal by ARG (Fig. 1A-1B).

The biodistribution of ^177^Lu-FAP-2287 was evaluated in vivo by SPECT/CT imaging following a single dose of 30 MBq ^177^Lu-FAP-2287 in MCA205-mFAP tumor bearing mice (n = 6). The radiotracer was rapidly enriched within the MCA205-mFAP xenografts with low off-target accumulation and predominantly bladder via renal elimination (Fig. 1C-E). High tumor to low background signal was observed as normal tissues such as kidney and liver showed very limited uptake. At 3 h pi, ^177^Lu-FAP-2287 accumulated in MCA205-mFAP tumors with 8.2% ID/g uptake, which was maintained at 2.9% ID/g by 24 h pi, respectively. The kidneys had the most non-target uptake of all the organs, with the highest level of 2.0% ID/g observed at 3 h pi resulting in the tumor-to-kidney (T/K) ratio 4.1-fold.

### Anti-tumor activity of ^177^Lu-FAP-2287 plus anti-PD-1 on syngeneic tumors

**Fig. 2:**
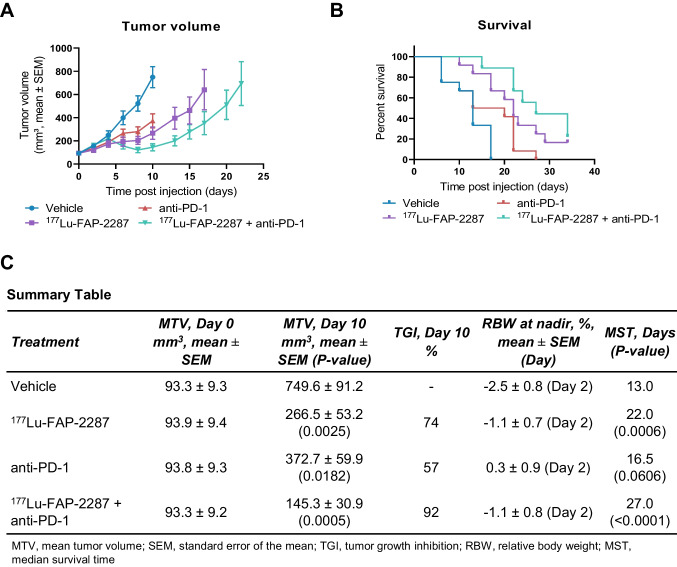
Tumor efficacy of ^177^Lu-FAP-2287 and in combination with anti-PD-1 in MCA205-mFAP syngeneic tumor model. MCA205-mFAP tumor-bearing mice were treated with vehicle, ^177^Lu-FAP-2287, anti-PD-1 antibody, or combination ^177^Lu-FAP-2287 plus anti-PD-1 (n = 12 mice/group). Mean tumor volumes ± SEM during study period (**A**), Kaplan Meier survival curve (**B**) and a summary table (**C**) are shown

The combinatory therapeutic effect of an anti-PD-1 antibody and ^177^Lu-FAP-2287 was evaluated in vivo in the MCA205-mFAP tumor model. Tumor bearing mice were treated with vehicle, ^177^Lu-FAP-2287 (60 MBq), anti-PD-1 (10 mg/kg) and the combination (n = 12 per group). On day 10 pi, tumors in the vehicle control animals reached an MTV of 750 mm^3^, while significant tumor growth inhibition (TGI) was observed in ^177^Lu-FAP-2287, anti-PD-1 and the combination group with MTV of 266, 373 and 145 mm^3^, corresponding to a TGI of 74, 57, and 92%, respectively (*P* < 0.05, Fig. 2A, C). A significant difference in MTV between combination ^177^Lu-FAP-2287 plus anti-PD-1 versus anti-PD-1 alone (*P* = 0.0247) was observed but not when compared to ^177^Lu-FAP-2287 alone (*P* = 0.2377). The median survival time (MST) was also delayed with ^177^Lu-FAP-2287 monotherapy and ^177^Lu-FAP-2287 plus anti-PD-1 combination resulting in MST of 22 and 27 days, respectively, compared to vehicle group survival of 13 days (*P* < 0.001, Fig. 2B-C).

### Immune profiling of MCA205-mFAP tumors treated with ^177^Lu-FAP-2287 and anti-PD-1 combination

The immune infiltration in response to ^177^Lu-FAP-2287 was investigated in a separate study. Mice bearing MCA205-mFAP tumors were dosed with vehicle, ^177^Lu-FAP-2287 (60 MBq), anti-PD-1 (10 mg/kg), and ^177^Lu-FAP-2287 plus anti-PD-1 (n = 8 per group). Tumors were analyzed by flow cytometry, IHC, and RNA microarray on days 8 and 13 pi. Tumor masses on day 13 pi with ^177^Lu-FAP-2287 plus anti-PD-1 treatment were smaller at 424 mg than vehicle controls which were 1433 mg (Supplementary Fig. S4A-S4B). In addition, ^177^Lu-FAP-2287 had a significant impact on tumor weight at 746 mg compared to vehicle control on day 13 pi (*P* = 0.0048).

**Fig. 3:**
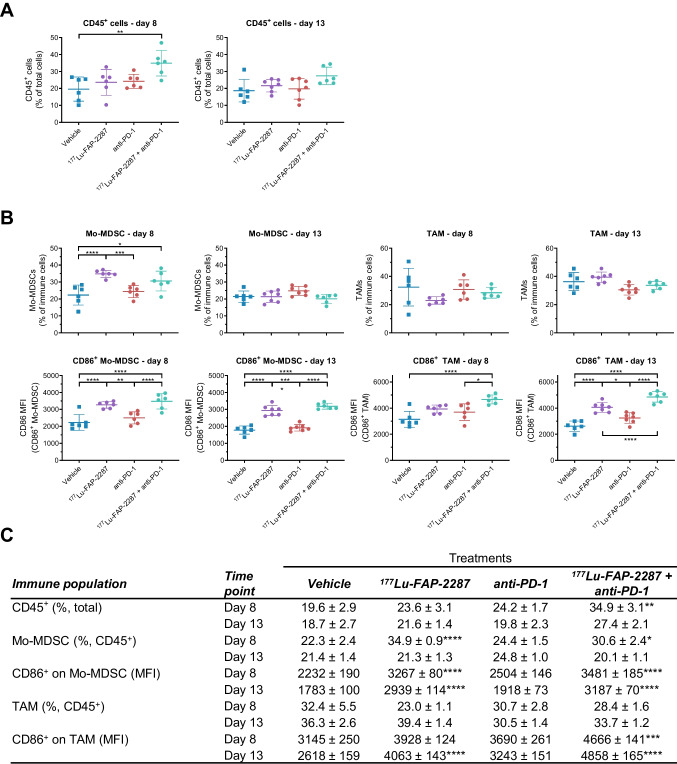
Immune profiling of CD86^+^ myeloid subsets after treatment with ^177^Lu-FAP-2287 and in combination with anti-PD-1 in MCA205-mFAP syngeneic tumor model. Individual percentage of CD45^+^ cells with bars as mean ± SEM on day 8 and 13 pi (**A**), individual percentage of Mo-MDSC and TAM and their CD86 MFI with bars as mean ± SEM on day 8 and 13 pi (**B**), and a summary table (**C**) are shown. Significant changes compared to vehicle in the summary table and between groups in the graph are denoted as * *p* < 0.05, ** *p* < 0.01, *** *p* < 0.001 and **** *p* < 0.0001

Surface marker analysis of immune cell populations in tumors by flow cytometry revealed more CD45^+^ leukocytes with the combination treatment of ^177^Lu-FAP-2287 plus anti-PD-1 at the earlier timepoint day 8 pi, where CD45^+^ cells increased to 34.9% of total cells compared to 19.6% and 23.6% for vehicle and ^177^Lu-FAP-2287 monotherapy, respectively (*P* < 0.05, Fig. 3A, C); though levels were comparable to vehicle by day 13 pi. Breakdown into subsets of immune cells on day 8 pi for the ^177^Lu-FAP-2287 monotherapy and ^177^Lu-FAP-2287 plus anti-PD-1 combination showed monocytic myeloid-derived suppressor cells (Mo-MDSCs) significantly increased from 22.3% of CD45^+^ cells for vehicle control to 34.9% and 30.6%, respectively (*P* < 0.05, Fig. 3B-C), while no changes in tumor associated macrophages (TAMs) were observed. Conversely, polymorphonuclear myeloid-derived suppressor cells (PMN-MDSCs) decreased from 21.7% in vehicle animals down to 5.4% for the combination (*P* = 0.0085, Supplementary Fig. S5A-S5B). Myeloid subsets were unaffected for all groups on day 13 pi, correlating with the lack of change in total CD45^+^ cells.

### CD86 activation of Mo-MDSCs and TAMs with ^177^Lu-FAP-2287 and anti-PD-1 combination

Staining for costimulatory marker CD86 showed that ^177^Lu-FAP-2287, both as single agent and combined with anti-PD-1, increased activation of myeloid subsets. Activated CD86^+^ Mo-MDSCs went from 75.9% and 70.6% of Mo-MDSCs in vehicle control on days 8 and 13 pi, to 84.9% and 82.2% with ^177^Lu-FAP-2287 treatment, respectively (*P* < 0.0001, Supplementary Fig. S5A-S5B). In addition, CD86 levels on Mo-MDSCs increased from 2232 and 1783 MFI in vehicle control on days 8 and 13 pi, respectively, to 3267 and 2939 MFI with ^177^Lu-FAP-2287 treatment (*P* < 0.0001, Fig. 3B-C). No difference was observed whether ^177^Lu-FAP-2287 was dosed as single agent or combined with anti-PD-1.

Similar to Mo-MDSCs but delayed till day 13 pi, CD86^+^ M1 polarized TAMs increased with ^177^Lu-FAP-2287 single agent and when combined with anti-PD-1. CD86^+^ TAMs went from 85.2% of TAMs in vehicle control to 92.0% with ^177^Lu-FAP-2287 therapy and showed no differences with ^177^Lu-FAP-2287 alone versus combined with anti-PD-1 (*P* = 0.0037, Supplementary Fig. S5A-S5B). Earlier and stronger CD86 induction in TAMs was measured with the ^177^Lu-FAP-2287 plus anti-PD-1 combination, with greater MFI of 4666 observed on day 8 pi versus 3145 MFI for vehicle control, which was maintained on day 13 pi at 4858 MFI compared to vehicle 2618 MFI (*P* < 0.0001, Fig. 3B-C). At the later timepoint, ^177^Lu-FAP-2287 alone also increased to 4063 MFI but was significantly lower than the combination ^177^Lu-FAP-2287 plus anti-PD-1.

### T cell tumor infiltration with ^177^Lu-FAP-2287 and anti-PD-1 combination

**Fig. 4:**
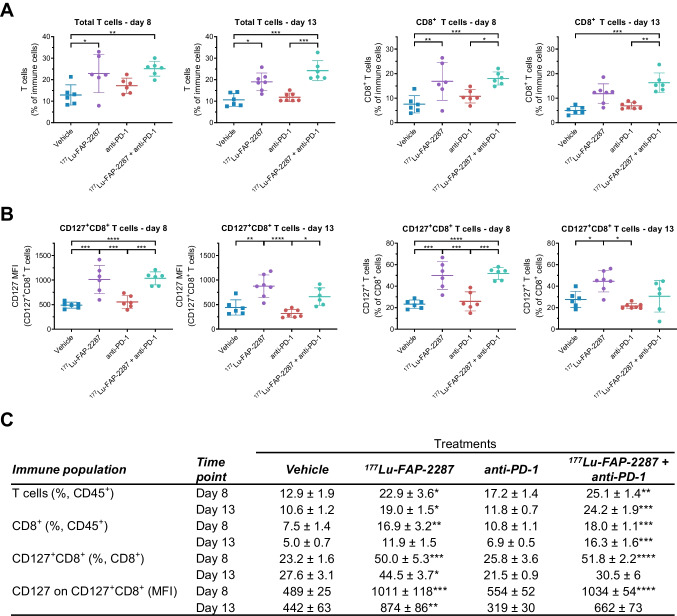
Immune profiling of CD8^+^ T cells after treatment with ^177^Lu-FAP-2287 and in combination with anti-PD-1 in MCA205-mFAP syngeneic tumor model. Individual percentage of T cells and CD8^+^ T cells with bars as mean ± SEM on day 8 and 13 pi (**A**), individual percentage of CD127^+^CD8^+^ cells and their CD127 MFI with bars as mean ± SEM on day 8 and 13 pi (**B**), and a summary table (**C**) are shown. Significant changes compared to vehicle in the summary table and between groups in the graph are denoted as * *p* < 0.05, ** *p* < 0.01, *** *p* < 0.001 and **** *p* < 0.0001

The T cell population expanded to 22.9% and 25.1% with ^177^Lu-FAP-2287 and ^177^Lu-FAP-2287 plus anti-PD-1 treatments, respectively, compared to vehicle control of 12.9% on day 8 pi (*P* < 0.05, Fig. 4A). On day 13 pi, ^177^Lu-FAP-2287 monotherapy and ^177^Lu-FAP-2287 plus anti-PD-1 maintained significantly higher levels of T cells with 19.0% and 24.2% of CD45^+^ cells, respectively, compared to 10.6% for vehicle. CD8^+^ T cell infiltration also increased on day 8 pi with ^177^Lu-FAP-2287 and ^177^Lu-FAP-2287 plus anti-PD-1 treatments, raising CD8^+^ T cell percentage to 16.9% and 18.0% of CD45^+^ cells, respectively, compared to 7.5% for vehicle control (*P* < 0.01, Fig. 4A). On day 13 pi, only the combination maintained significant elevated CD8^+^ T cells with 16.3% compared to 5.0% in vehicle animals (*P* = 0.0002). In addition, ^177^Lu-FAP-2287 augmented the levels of the memory-associated marker CD127 on CD8^+^ T cells, with levels increasing on day 8 pi to 50.0% of CD8^+^ T cells and 1010 MFI, respectively, from 23.2% and 489 MFI for vehicle control and remaining effective on day 13 pi with 44.5% of CD8^+^ cells and 874 MFI, respectively, compared to 27.6% and 442 MFI for vehicle control (*P* < 0.05, Fig. 4B). Treatments had minimal effects on CD25^+^CD8^+^ subset and CD25 MFI levels in CD8^+^ T cells (Supplementary Fig. S6). No changes were observed in CD4^+^ T cells, including CD25^+^ and CD127^+^ subsets with any treatments when compared to vehicle (Supplementary Fig. S7), suggesting that changes in total T cells are mostly due to modulations in CD8^+^ T cells.

**Fig. 5:**
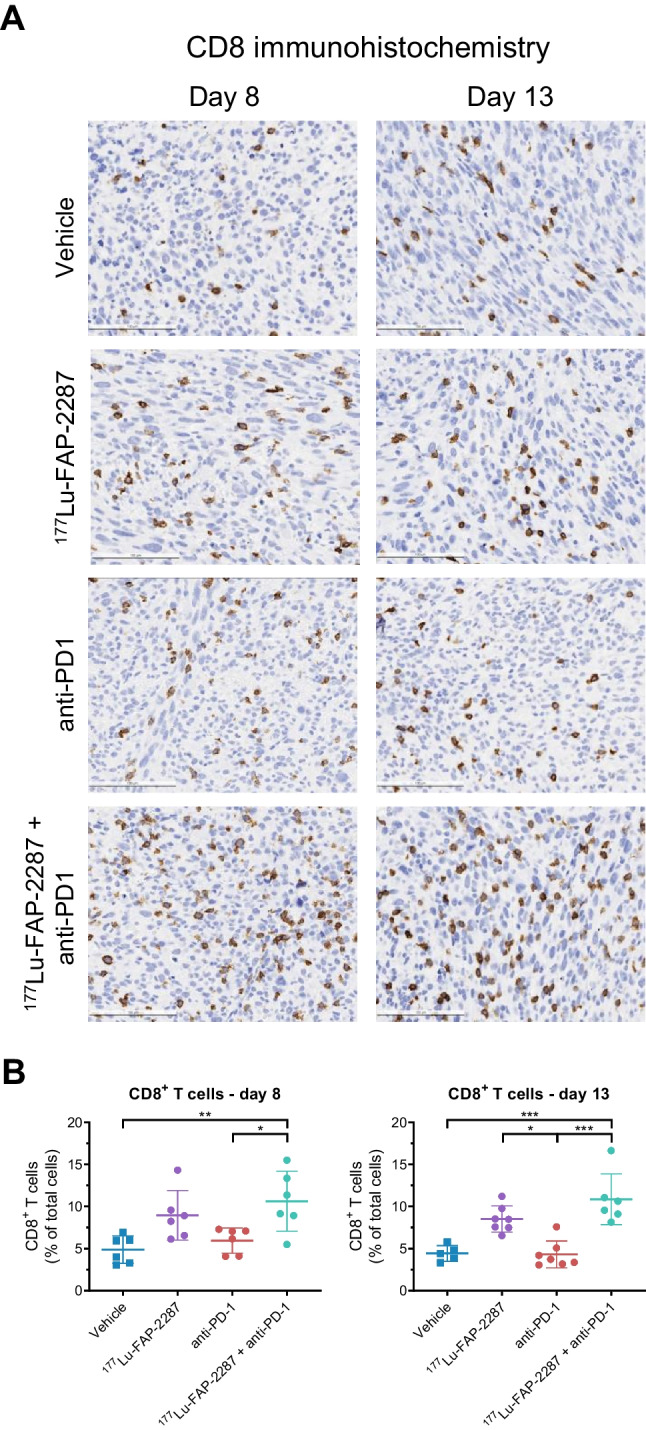
IHC analysis of CD8^+^ T cells after treatment with ^177^Lu-FAP-2287 and in combination with anti-PD-1 in MCA205-mFAP syngeneic tumor model. Representative images show CD8 staining in MCA205-mFAP tumors (**A**, 100 μm) and individual percentage of CD8^+^ cells by IHC quantification with bars as mean ± SEM are plotted for days 8 and 13 pi (**B**). Significant changes between groups are denoted as * *p* < 0.05, ** *p* < 0.01, *** *p* < 0.001 and **** *p* < 0.0001

IHC analysis confirmed higher levels of CD8^+^ T cell tumor infiltrates with the combination ^177^Lu-FAP-2287 plus anti-PD1 on day 8 pi, with the number of CD8^+^ T cells increasing to 10.6% of total cells versus to 4.9% (*P* = 0.0018) and 6.0% (*P* = 0.0190) in vehicle and anti-PD-1 groups, respectively (Fig. 5A-B). In addition, higher levels were observed in ^177^Lu-FAP-2287 treated tumors with 9.0% CD8^+^ T cells but was not significant. Similar results were obtained on day 13, with increased CD8^+^ T cell infiltrates for the combination with 10.9% CD8^+^ T cells compared to 4.4% (*P* = 0.0007) and 4.3% (*P* = 0.0002) in vehicle and anti-PD-1 groups, respectively. Elevated levels were also observed in ^177^Lu-FAP-2287 treated tumors with 8.5% CD8^+^ T cells which was significantly different than anti-PD-1 (*P* = 0.0249) but not to vehicle or the combination groups. IHC analysis of CD4^+^ T cells corroborated flow cytometry results, showing nonsignificant differences between groups on day 8 pi with CD4 levels ranging from 2.9% to 4.1%; and on day 13 from 3.0% to 4.0% (Supplementary Fig S8A-S8B).

### Upregulation of STING pathway inducible genes in MCA205-mFAP tumors after treatment with ^177^Lu-FAP-2287 and anti-PD-1 in vivo

**Fig. 6:**
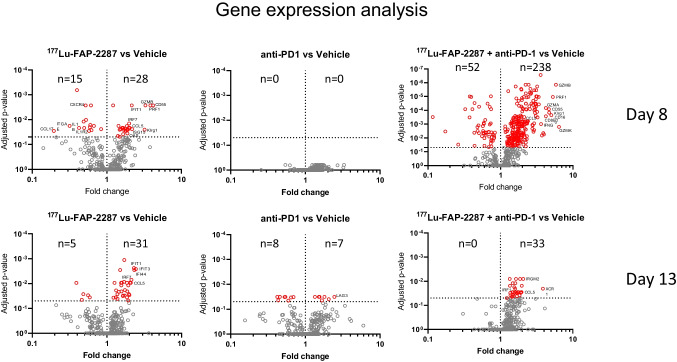
Differential expression analysis of MCA205-mFAP tumors after treatment with ^177^Lu-FAP-2287 and in combination with anti-PD-1. Fold changes in gene expression versus vehicle are shown as volcano plots with statistically significant genes above the horizontal line, and highly differentially expressed genes on either side

Molecular profiling of the immune system was performed using a multiplex RNA hybridization assay with the PanCancer Immune Profiling Panel, a 770-plex gene array including 750 genes to assess mouse immune responses and 20 internal reference genes. RNA levels were measured in MCA205-mFAP tumors treated with vehicle, ^177^Lu-FAP-2287 (60 MBq), anti-PD-1 (10 mg/kg), and ^177^Lu-FAP-2287 plus anti-PD-1. On day 8 pi, differential expression analysis to vehicle control showed ^177^Lu-FAP-2287 significantly increased RNA levels of 28 genes with 16 involved in interferon response including ISG15, IFIT1, CCL5, OAS3 and IRF7, and others in natural killer cell biology including KLRG1, GZMB and PRF1; while 15 gene transcripts were decreased such as CCL17 and CXCR4 (Fig. 6). The combination with anti-PD-1 had more dramatic changes in RNA expression compared to ^177^Lu-FAP-2287 monotherapy with transcript levels of 238 genes increasing and 52 decreasing. These included all those reported with single agent ^177^Lu-FAP-2287 except for IL1B, IL1R2, and ITGAE. No significant fold changes were observed for the anti-PD-1 monotherapy.

Comparison of fold changes to vehicle control for differentially expressed genes (that were statistically significant) shared between single agent ^177^Lu-FAP-2287 versus the combination with anti-PD-1 treatment showed direct correlation with similar induction or reduction levels (*r* = 0.9709, Supplementary Fig. S9A-S9B), suggesting that the addition of anti-PD-1 modifies the scope of the immune molecular profile but not the intensity. Changes in tumor RNA expression were maintained by day 13 pi with ^177^Lu-FAP-2287 monotherapy so that 16 of the 43 (84%) RNA transcripts originally identified at day 8 pi still remained significantly differentially expressed, including 13 genes involved in the interferon signaling pathway. The reduction in differential gene expression was pronounced in tumors from the combination treatment animals at day 13 pi, with only 33 of the original 238 (11%) genes identified at day 8 still showing differential expression, despite continual anti-PD-1 dosing.

From the RNA expression data, gene set analysis was performed using global significance statistics (GSS) to assess immune functions with the most profound changes, through an averaging of the significance measures across all the genes in the pathway. GSS score for the interferon signaling pathway was the highest for the monotherapy ^177^Lu-FAP-2287 compared to vehicle. The combination treatment in general had high GSS scores across all gene sets, with the highest changes observed in the natural killer cell functions. Several other immune processes were associated with high GSS including T cell, macrophage, dendritic cell and MHC functions (Supplementary Fig. S9C).

**Fig. 7:**
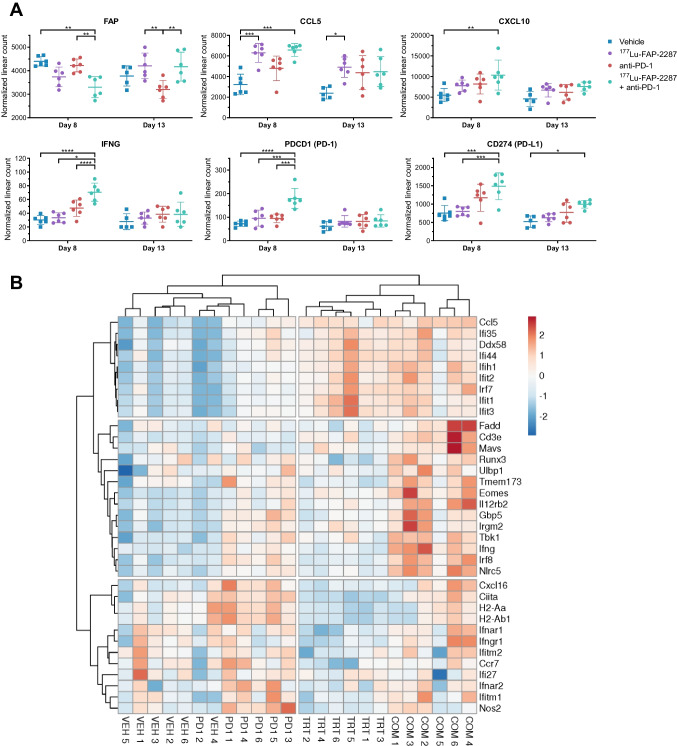
Molecular profiling of MCA205-mFAP tumors after treatment with ^177^Lu-FAP-2287 and in combination with anti-PD-1. Individual normalized linear count with bars as mean ± SEM are plotted for FAP and several immune genes on days 8 and 13 pi (**A**). Significant changes between groups are denoted as * *p* < 0.05, ** *p* < 0.01, *** *p* < 0.001 and **** *p* < 0.0001. The heatmap was obtained by hierarchical clustering centered around genes with correlation distance and complete linkage, using ClustVis tools (https://bio.tools/clustvis). The color scale displays the row z-score: red color indicates high abundance while blue color low abundance (B). Groups are marked as VEH, vehicle; TRT, ^177^Lu-FAP-2287; PD1, anti-PD-1, COM, combination

Individual gene expression analysis of tumors after treatments confirmed several STING pathway regulated genes were altered after exposure to radiation delivered by ^177^Lu-FAP-2287. MCA205-mFAP tumors increased CCL5 by 1.95 and 2.04-fold in response to ^177^Lu-FAP-2287 and ^177^Lu-FAP-2287 plus anti-PD-1 combination, respectively (Fig. 7A). However, CXCL10 and IFNG required the addition of anti-PD-1 to be induced to 1.89 and 2.34-fold with ^177^Lu-FAP-2287 plus anti-PD-1 combination, suggesting radiation alone was insufficient and synergy between the two therapeutics caused the rise in expression. Activation of the IFN regulated genes consequently showed better induction with the combination including its inducible genes PD-1 (PDCD1) and PD-L1 (CD274) which were 2.49 and 1.96-fold, respectively (Fig. 7A). In addition, FAP RNA expression in MCA205-mFAP tumors were lower in combination group on day 8 pi, while by day 13 pi, FAP was substantially lower in anti-PD-1 treated tumors than with ^177^Lu-FAP-2287 either alone or with anti-PD-1.

Expression of additional interferon pathway genes were evaluated for response to ^177^Lu-FAP-2287, anti-PD-1 and ^177^Lu-FAP-2287 plus anti-PD-1 combination in the MCA205-mFAP treated tumors. The heatmap generated using expression data for day 8 pi showed hierarchical clustering of ^177^Lu-FAP-2287 samples with those of ^177^Lu-FAP-2287 plus anti-PD-1 combination (Fig. 7B). Several genes identified previously by differential expression analysis grouped with CCL5 such as IFIT1 and IRF7 and had increased levels in tumors treated with ^177^Lu-FAP-2287 as single agent or in combination with anti-PD-1. However, another subset of genes showed higher expression in the combination treated tumors than with either monotherapy, including IFNG, the T cell receptor subunit CD3E, and STING pathway component TBK1. And the last gene subgroup appeared to be more involved with anti-PD-1 efficacy than with ^177^Lu-FAP-2287, in particular CIITA, H2-Aa and H2-Ab1 which are part of the MHC complex.

### Activation of the STING pathway in MCA205-mFAP cells in vitro

The activation of the STING signaling pathway in MCA205-mFAP cells was verified using external beam radiation therapy (EBRT) in vitro. First, the EBRT cytotoxic effect on cell viability was assessed after 5 days, and radiation IC_50_ value of 10 Gy was determined to inhibit 50% of MCA205-mFAP cell proliferation (Supplementary Fig. S10A). Induction of CCL5 and CXCL10 expression through STING activation was examined next in MCA205-mFAP 3 days after treatment with 10 Gy EBRT. RNA analysis by qPCR showed 17.8 and 5.5-fold upregulation of CCL5 and CXCL10 RNA in MCA205-mFAP cells compared to non-irradiated control, respectively (Supplementary Fig. S10C). Knockdown of STING pathway proteins cGAS, STING1, IRF3, and TBK1 by siRNA transfection reduced expression of gene targets by greater than 0.4-fold (range 0.04–0.42; Supplementary Fig. S10B). And siRNA transfected cells had diminished CCL5 and CXCL10 upregulation, down to levels comparable with non-irradiation controls, with one exception of TBK1 partially reverting to untreated levels of CCL5. IFNG RNA was not detected in the in vitro cultured cells (data not shown). Supernatant from cultured cells were also examined for CCL5 and CXCL10 chemokines secreted by MCA205-mFAP cells and had increased levels of 13.9 and 12.4-fold respectively with EBRT, which were reduced with siRNA knockdown of cGAS, STING1, IRF3, and TBK1 genes to nontreated levels (Supplementary Fig. S10D).

## Discussion

The current study provides insights into the immunomodulatory functions of FAP-targeted radiotherapy and demonstrates the therapeutic potential of combining ^177^Lu-FAP-2287 with an anti-PD-1 monoclonal antibody in a mouse sarcoma model. FAP-2287 was used as a murine substitute for the clinical molecule FAP-2286 due to its comparable affinity for murine FAP (K_D_ = 0.5 ± 0.1 nM) versus FAP-2286 for human FAP (K_D_ = 0.4 ± 0.2 nM) when both are chelated with lutetium. In contrast to its high tumor retention in xenograft tissues and in patients [16, 22], FAP-2286 is not ideally suited for targeting FAP in syngeneic mouse models due to its substantial lower affinity towards murine FAP (K_D_ = 3.8 ± 1.0 nM) [16]. Therefore, FAP 2287 was utilized in this study in an attempt to replicate FAP-2286 biological activity in human.

Mechanistically, the results suggest that from an immunological perspective, DNA fragmentation induced by damage from radiation emitted from ^177^Lu-FAP-2287 or EBRT activates the cGAS-STING pathway resulting in the induction of genes encoding type I interferons as exemplified by IFNG and the CD8^+^ T cell attracting chemokines CCL5 and CXCL10 in MCA205-mFAP tumors following ^177^Lu-FAP-2287 administration. Consistent with the transcriptome changes, corresponding alterations in the immune cell populations towards an adaptive anti-tumor immune response within the intratumoral compartments were also demonstrated including an increase in CD8^+^ T cells coupled with CD86^+^ activated Mo-MDSCs and TAMs, ultimately leading to an anti-tumor immune response.

Previous preclinical reports have also demonstrated the combined use of ICI together with TRT leads to substantial suppression of tumor growth with an improvement in survival [13, 23–25]. The improved response to the combination was attributed to an increase in tumor infiltrating CD8^+^ T cells, and the generation of immunologic memory T cells, as rechallenged mice rejected implanted cancer cells [13, 23–25]. However, the impact of TRT on CD4^+^ T cells appears unsettled as concurrent changes with CD4^+^ T cells have either decreased or remained unaltered as observed in this study and others [26, 27]. In opposition to increasing CD8^+^ T cells, the CD11b^+^ myeloid population as a whole decreased with ^177^Lu-FAP-2287, most appreciably the PMN-MDSCs and, to a lesser extent, the TAMs while the Mo-MDSCs increased, suggesting differences in TRT mediated immunomodulatory effects on myeloid lineages and their responses. Interestingly, additional examination into TAMs and Mo-MDSCs subsets showed upregulation of the costimulatory marker CD86 in these cells, indicating polarizing towards a pro-inflammatory, anti-tumorigenic phenotype with antigen presenting and stimulation capacities [28]. Others have noted increased CD11b^+^ cells with TRT but have not researched their functions or subpopulations [13, 26]. Further dissection of CD11b^+^ cell subsets is necessary to better understand their role and impact on the immune response.

Despite strong activation by TRT, activated immunity can quickly be dampened if suppressive signals are present that prevent anti-tumor immune responses. In the MCA205-mFAP model, tumor inhibition with combination ^177^Lu-FAP-2287 plus anti-PD-1 was observed on day 8 pi with heightened immune responses but by day 13 pi tumor control was lost perhaps in part reflecting immune pro-inflammatory signaling subsiding. In parallel, DNA damage and tumor cell death induced by beta-particle radiation emitted by ^177^Lu-FAP-2287 is essentially absent at day 13, further reducing anti-tumor effects.

One significant limitation of the current study is the lack of a preclinical tumor model that reflects the FAP-positive CAF abundance, localization and biology that are observed in human tumors. Previous reports have demonstrated that in many tumor types CAFs with high FAP expression are enriched in the TME [16]. In cancers of epithelial origin, FAP expression is mainly restricted to the CAFs in the TME, which possesses diverse roles in cancer biology, such as stromatogenesis, reciprocal signaling interactions with cancer cells, and immune suppression through crosstalk with tumor-infiltrating leukocytes [29–31]. By cleaving factors relevant to TME, FAP-positive CAFs can shape the immunosuppressive TME through modifying its distinct cytokines [32]. FAP-targeted radiotherapy would not only impact immunosuppressive FAP-positive CAFs but also affect neighboring cancer and stromal cells in the TME through crossfire effects given the distance travelled by the average beta-particle emission from ^177^Lu (~ 100 cell diameters) [33]. This indirect cytotoxicity of cancer cells may induce a direct immune response to cancer cells as has been shown in this study using a syngeneic model of FAP-expressing sarcoma. Additional work in immunocompetent mouse tumor models developing more translationally relevant FAP expressing CAFs is warranted to further investigate the potential of beta-emitters in targeting the TME.

To be an effective immunostimulant, the biodistribution of TRT agents should be restricted to tumors and with limited uptake in normal organs, especially those involved with the generation of an effective immune response such as the lymphatic system. This approach minimizes the potentially negative impact of radiation on immune function and the prevention of hematological toxicities. In previous reports studying the TRT ^177^Lu-NM600 and ^90^Y-NM600, bone marrow was the dose-limiting organ and showed signs of a dose-dependent radiotoxicity as evidenced by transient lymphopenia and anemia [15, 25]. In addition, transient blood cytopenia have been reported with TRT treatments resulting in reduced bone marrow and spleen cellularity. However, robust antitumor efficacy including immune cell activation were maintained indicating the effects of TRT on systemic versus antitumor immunity may differ [15, 34]. The studies described presently were not designed to assess the long-term hematologic toxicities including secondary malignancies that have been reported in patients with targeted radiotherapies [35], and further investigations into TRT actions on hematology are warranted.

## Conclusions

In a mouse model of sarcoma, FAP-targeted radionuclide therapy induces an immunogenic TME through infiltration and activation of immune cells resulting in enhanced tumor efficacy when combined with PD-1 checkpoint inhibition. These findings provide a rationale for clinical studies of combined ^177^Lu-FAP-2286 radiotherapy and immune checkpoint inhibition in FAP-positive tumors.

## Supplementary Information

Below is the link to the electronic supplementary material.

Supplementary file1 (DOCX 55 KB)

Supplementary file2 (PDF 1035 KB)

## Data Availability

The data generated in this study are available within the article and its supplementary data files.
