# Supplementary material for: Fibroblast activation protein targeted radiotherapy induces an immunogenic tumor microenvironment and enhances the efficacy of PD-1 immune checkpoint inhibition
Source: Eur J Nucl Med Mol Imaging. 2023 Apr 22;50(9):2621–35. doi: 10.1007/s00259-023-06211-6 (PMC10317891; doi:10.1007/s00259-023-06211-6)
Supplement: Supplementary file 1 — Supplementary file1 (DOCX 55 KB) [file 259_2023_6211_MOESM1_ESM.docx]

**Fibroblast activation protein targeted radiotherapy induces an immunogenic tumor microenvironment and enhances the efficacy of PD-1 immune checkpoint inhibition**

Dirk Zboralski^1^, et al

^1^3B Pharmaceuticals GmbH, Berlin, Germany; ^2^Minerva Imaging ApS, Ølstykke, Denmark; ^3^Clovis Oncology, Inc., Boulder, Colorado, USA

**Corresponding Author:** Dirk Zboralski, 3B Pharmaceuticals GmbH, Magnusstraße 11, D-12489 Berlin, Germany; Phone: +49-30-63924317; E-mail: dirk.zboralski@3b-pharma.com

# Supplementary Materials and Methods

**Synthesis of FAP-2287**

To generate FAP-2287, the peptide H-Cys-Pro-Pro-Thr-Gln-Phe-Cys-Asp-NH2 was assembled according to the 'General procedures for Automated/Semi-automated Solid-Phase Synthesis' in a 50 umol scale on a Rink amide resin. The lyophilized linear peptide was dissolved in 60 ml of a 1:1 mixture of ammonium bicarbonate solution (50 mM, pH = 8.5) and acetonitrile. To this mixture a solution of 26.8 mg 1,3,5-tris(bromomethyl)benzene (75 mmol, 1.5 eq compared to initial resin loading) in 0.5 ml acetonitrile was added. After stirring the solution for 1 hour 43 mg piperazine (500 mmol, 10 eq compared to initial resin loading) were added. After 2 hours 50 ml TFA were added and the solvent removed by lyophilization. The remainder was subjected to HPLC purification (15 to 45% B in 30 min - Kinetex) to yield 9.15 mg (7.4 mmol) of the peptide intermediate Hex-[Cys(tMeBn(H-PP))-Pro-Pro-Thr-Gln-Phe-Cys]-Asp-NH2 (14.7%). To the solution of the latter in 150 ml DMSO 2.5 ml DIPEA were added to adjust the pH value to approximately 7.5 - 8. Then 8.4 mg of DOTA-NHS (11 mmol, 1.5 eq compared to the peptide intermediate) in 100 ml DMSO were added. During the course of the LC/TOF-MS monitored reaction 2.5 ml DIPEA was added 3 times to re-adjust the pH value to the starting value. After reaction completion the solution was subjected to HPLC purification (15 to 45% B in 30 min - Kinetex) to yield 7.09 mg of the pure title compound (8.7% overall yield). HPLC: tR = 6.0 min. LC/TOF-MS: exact mass 1628.706 (calculated 1628.704).

**Immunohistochemistry**

A tissue slide containing both intrinsic positive staining structures was included in every staining run. For FAP H-score assessment, the staining intensity was scored as 0, 1, 2, or 3 corresponding to the presence of negative, weak, intermediate, and strong brown staining, respectively. The average percentage positive was calculated for each intensity category and the following calculation was applied: H-score = (% of cells stained at intensity category 1 x 1) + (% of cells stained at intensity category 2 x 2) + (% of cells stained at intensity category 3 x 3). Image analysis scoring was previously validated against scoring performed by a pathologist. For CD4 and CD8 percentage assessment, QuPath positive cell detection function was used.

**Autoradiography**

Autoradiographic evaluation was performed on 20 µm thick sections attached to chrome-gelatin coated slides. ^111^In-labeled FAP-2287 was diluted in incubation buffer (0.17 M Tris, pH 8.2, 1% bovine serum albumin [BSA]) to the desired radioactivity concentration (2 × 10^5^ counts per minute [CPM]/mL), which was verified via gamma counting. In order to assess nonspecific binding, an adjacent slide was incubated in tracer solution admixed with 1 µM of unlabeled FAP-2287–related blocking peptide. After 2-hour incubation at room temperature, the slides were washed 4 times for 5 minutes in precooled wash buffer 1 (0.17 M Tris, pH 8.2, 0.25 % BSA) and 2 times in wash buffer 2 (0.17 M Tris, pH 8.2), before drying the sections for at least 30 minutes. Protein-specific binding was analyzed with a CCD camera system and the corresponding MCID analysis software (Interfocus, RRID:SCR_014278). Signal strength was measured once on the tissue with total tracer binding and once in the same region of interest on the sample for nonspecific binding. For the evaluation of every experiment, a separate standard curve (100–100,000 CPM) was recorded and tissue-bound radioactivity was calculated based on the resulting power equation using GraphPad Prism 8.4 (RRID:SCR_002798).

**Biacore assay**

Biacore CM5 sensor chips (GE Healthcare Life Sciences) were used for surface plasmon resonance (SPR) studies. Human FAP antibody (MAB3715, R&D systems) or mouse FAP antibody (MAB97271, R&D systems) was diluted in 10 mM acetate buffer, pH 4.5, to a final concentration of 50 µg/mL. Antibodies were directly immobilized onto the flow cells at 25°C using the Amine Coupling Kit Reagent solutions (GE Healthcare Life Sciences), according to the manufacturer’s recommendations. A preinstalled program for immobilization was used with an immobilization level of 7000 RU, flow rate of 10 µL/minute.

Recombinant human FAP (rhFAP; Sino Biological) and mouse FAP (rmFAP; R&D systems) were diluted in Running Buffer (150 mM NaCl, 10 mM HEPES, 0.005% TWEEN-20, pH 7.4; HBST) to a final concentration of 20 and 4 µg/mL, respectively. A five-fold dilution series of test compounds with concentrations ranging from 50 nM to 0.19 nM was injected over the sensor surface for 120 seconds at a flow rate of 30 µl/minute. The dissociation was monitored for 1800 seconds and the surface was regenerated with 10 mM glycine pH 2. SPR binding analyses for binary complexes were performed in single cycle kinetics (SCK) mode at 25°C. Following 3 SCK measurements, a baseline drift was assessed by injecting running buffer through a flow cell, with the antibody and FAP immobilized to the sensor surface.

For each test compound, SPR raw data in the form of resonance units (RU) were plotted as sensorgrams using the Biacore T200 control software (RRID:SCR_019718). The signal from the blank sensorgram was subtracted from that of the test compound sensorgram (blank corrected). The blank corrected sensorgram was corrected for baseline drift by subtracting the sensorgram of a SCK run without the test compound (running buffer only). The association rate (k_on_), dissociation rate (k_off_), dissociation constant (K_D_), and t_1/2_ were calculated from Blank-normalized SPR data using the 1:1 Langmuir binding model from the Biacore T200 evaluation software (GE Healthcare Life Sciences).

**Cell-based binding assay**

In order to determine binding of FAP-2287 and metal-chelates to FAP-expressing cells, a competitive fluorescence-activated cell sorting (FACS) binding assay was established. FAP-expressing human WI-38 fibroblasts (obtained from the European Collection of Authenticated Cell Cultures, ECACC, RRID:CVCL_0579) were cultured in Eagle’s Minimum Essential Medium (EMEM) including 15% fetal bovine serum, 2 mM L-Glutamine, and 1% nonessential amino acids and used within 20 passages. WI-38 cells were tested negative for mycoplasma contamination (Minerva Analytix GmbH) and were authenticated by ATCC cell line authentication service in July 2021. Cells were detached with Accutase (Biolegend, #BLD-423201) and washed in FACS buffer (PBS including 1% FBS). Cells were diluted in FACS buffer to a final concentration of 100,000 cells/mL and 200 µL of the cell suspension were transferred to a U-shaped bottom, polypropylene 96-well plate (Greiner). Cells were washed in ice-cold FACS buffer and incubated with 3 nM of FAP-2287-related C-terminally Cy5-labeled competitor peptide in the presence of various concentrations of FAP-2287 and its metal chelates at 4°C for 1 hour. Cells were washed twice with FACS buffer and resuspended in 200 µL FACS buffer. Cells were analyzed in an Attune NxT flow cytometer (Thermo Fisher Scientific). Median fluorescence intensities (Cy5 channel) were calculated by Attune NxT software (RRID:SCR_019590) and plotted against peptide concentrations. Four parameter logistic (4PL) curve fitting and IC_50_/pIC_50_ calculations were performed using ActivityBase software (IDBS, RRID:SCR_004077).

**Protease activity assay**

Recombinant human FAP (R&D Systems, # 3715-SE) or mouse FAP (R&D Systems, # 8647-SE) was diluted in assay buffer (50 mM Tris, 1 M NaCl, 1 mg/mL BSA, pH 7.5) to a concentration of 3.6 nM. 25 µL of the FAP solution was mixed with 25 µL of a 3-fold serial dilution of the test compounds and incubated for 5 minutes in a white 96-well ProxiPlate (Perkin Elmer). As the specific FAP substrate, the FRET-peptide HiLyteFluor 488 - VS(D-)P SQG K(QXL 520) - NH2 was used (Eurogentec, Bainbridge, et al., Sci Rep, 2017, 7: 12524). 25 µL of a 30 µM substrate solution, diluted in assay buffer, was added. All solutions were equilibrated at 37°C prior to use. Substrate cleavage and increase in fluorescence (excitation at 485 nm and emission at 538 nm) was measured in a kinetic mode for 5 minutes at 37°C in a SpectraMax M5 plate reader (Molecular Devices). RFU/sec was calculated by SoftMax Pro software (RRID:SCR_014240) and plotted against peptide concentration. 4PL curve fitting and IC_50_/pIC_50_ calculations were performed using ActivityBase software (IDBS).

To test selectivity of FAP binding peptides toward both PREP and DPP4, protease activity assays were performed similarly to the FAP activity assay with following exceptions. PREP activity was measured with recombinant human PREP (R&D Systems, #4308-SE). As substrate 50 µM Z-GP-AMC (Bachem, # 4002518) was used. The DPP4 activity assay was performed in DPP assay buffer (25 mM Tris, pH 8.0), with recombinant human DPP4 (R&D Systems, #9168-SE). 20 µM of GP-AMC (Santa Cruz Biotechnology, #115035-46-6) was used as substrate. Fluorescence of AMC (excitation at 380 nm and emission at 460 nm) after cleavage was measured in a kinetic mode for 5 minutes at 37°C in a SpectraMax M5 plate reader (Molecular Devices).

**Plasma stability**

FAP-2287 was incubated in 50 µL of plasma with 3.8% sodium citrate as anticoagulant at a concentration of 10 µM. After 1, 2, 4, and 24 hours incubation at 37°C a suitable internal standard was added. FAP-2287 with plasma proteins were precipitated using 250 µL of a precipitation agent consisting of 1% trifluoroacetic acid in acetonitrile. Precipitation was carried out at room temperature for 30 minutes. After centrifugation at 18,200 relative centrifugal force (rcf) for 5 minutes, 100 µL of the supernatant was mixed with 100 µL of 1% formic acid.

A nonincubated plasma sample spiked with 10 µM sample concentration was prepared in the same way and was used as the reference. A pure plasma sample was incubated for 24 hours as a blank sample. A further pure plasma sample was incubated for 24 hours, processed as described above and spiked directly before analysis to determine the recovery. The samples were analyzed using an Agilent 1290 UHPLC system coupled to an Agilent 6530 QTOF mass spectrometer.

Chromatographic separation was carried out on a Phenomenex Aeris Peptide XB-C18 stationary phase (50 × 2.1 mm, 1.7-µm particle size) using a linear gradient from 2% B to 41% B in 7 minutes with 0.1% formic acid as eluent A and acetonitrile as eluent B. Flow rate was 0.8 mL/min and the column was heated to 40°C. Mass spectrometry detection was performed in positive ion mode. Complete mass spectra were collected for the whole chromatographic in a range between m/z 100 and 300 with a sampling rate of 3 spectra/second.

For the quantitative data analysis, the Agilent Quantitative Analysis B.08.00 software (RRID:SCR_015040) was used. The ion currents of the triply charged proton adducts of the compounds were extracted with a mass window of ±50 parts per million and the chromatographic peak was integrated. The concentration of the compound was determined by external matrix calibration with internal standard using 6 calibration samples (1, 2.5, 5, 7.5, 10, and 12.5 µM). A quadratic regression was performed. Carryover was determined by analyzing the blank sample directly after the highest calibration sample and comparing the determined peak areas. Recovery was calculated as the ratio between the spiked concentration of the recovery sample and the concentration calculated from the peak area of this sample.

**^177^Lu radiochemistry**

0.3–1.2 GBq ^177^LuCl_3_ (in 0.04 M HCl) were mixed with buffer (1 M sodium acetate/ascorbic acid buffer pH 5, containing 25 mg/mL methionine for FAP-2287 labeling; 100 µL per GBq ^177^LuCl_3_). 1 nmol of FAP-2287 (500 µM stock solution in water) per 30–60 MBq ^177^LuCl_3_ was added. The mixture was shaken at 300 – 600 rpm, 90°C for 15–25 minutes. After cooling down, DTPA and TWEEN-20 were added at a final concentration of 0.2 mM and 0.1%, respectively. In some instances, purification by solid phase extraction utilizing a C18 SepPak column pre-conditioned with absolute ethanol (5 mL) and water (10 mL) was performed. The crude product was diluted with water to a total volume of 5 mL, trapped on the pre-conditioned column, washed with water (5 mL) and then eluted with 500 μL 50/50 ethanol/water (v/v). The final product was formulated with 0.9% sterile NaCl for injection containing 10 mg/mL ascorbic acid to a final ethanol concentration of < 10%.

For assessment of labeling efficacy by TLC, an aliquot of the labeling solution was applied to a strip of iTLC-SG chromatography paper (Agilent) and developed in 0.1 M citric acid pH 5.4. The iTLC strip was then analyzed with a TLC scanner (Raytest). The radioactivity measured at the origin represents radiolabeled peptide, whereas the radioactivity at the solvent front represents free radionuclide. The radiochemical purity was analyzed by HPLC by injecting labeling solution onto either a Poroshell SB-C18 2.7 μm column (Agilent); eluent A: water, 0.1% TFA, eluent B: acetonitrile; gradient from 5% B to 70% B within 15 minutes, flow rate 0.5 mL/min; detector: NaI (Raytest), DAD 230 nm or a XBridge C18 3.5 μm, 4.6x50 mm column; eluent A: water, 0.1% TFA, eluent B: acetonitrile, 0.1% TFA; gradient from 5% B to 20% B within 1 minute, then from 20% B to 50% B within 7 minutes; flow rate 1.5 mL/min; detector: NaI (Raytest), DAD 220 nm. The peak eluting with the dead volume represents free radionuclide, the peak eluting with the peptide-specific retention time as determined with an unlabeled sample represents radiolabeled compound. Radiochemical purity was ≥90% at the end of synthesis.

**Animal housing and handling**

Studies with ^177^Lu labeled FAP-2287 and anti-PD-1 antibody were performed at Minerva Imaging ApS, Denmark using female C57BL/6 mice (Charles River Laboratories, Germany). For all experiments animals were ordered with an age of approximately 6 weeks. Animals were allowed an acclimatization period of at least one week prior to inoculation. Animals were housed in an animal room with 12 hours light and 12 hours darkness with free access to food and water. A maximum number of 5 animals were housed in the same cage, cages were equipped with nesting material and plastic or paper houses for hiding. Each cage was labeled with study ID, group and animal numbers and test compound. For efficacy studies animals were randomized according to tumor size. Animals were checked daily for any signs of illness or abnormal behavior. Main humane endpoints were defined as tumor volumes >1500 mm^3^, tumor burden >10% of animal weight, body weight loss >15% and tumor ulceration. When reaching one of these criteria the animal was sacrificed by cervical dislocation. All animal experiments were approved by the National Animal Experiments Inspectorate under the Ministry of Environment and Food of Denmark.

***In vivo* biodistribution**

Imaging and image analysis were performed as described. During the scan, mice were kept under isoflurane anesthesia, and body temperature was maintained under warm air. The following acquisition parameters were used for the respective imaging modality.

NanoSPECT/CT (Mediso), ^177^Lu-Imaging

| **Aquisition parameters SPECT** | | | |  |
| --- | --- | --- | --- | --- |
| Animals per bed | 3 | | | |
| Type of SPECT scanning | Multi-pinhole | | | |
| Time per projection | 30s (3h), 60s (24h and 48h), 120s (72h) | | | |
| Energy windows (keV) | Peaks (keV) | | Full width (%) | |
|  | Primary | 208.4 | 20% | |
|  | Secondary | 112.9 | 20% | |
|  | Tertiary | 56.1 | 20% | |
| **Aquisition parameters CT** | | | |  |
| Animals per bed | 3 | | | |
| Type of scan | Helical | | | |
| Projections | 480 | | | |
| Pitch | 1 | | | |
| X-ray power (kVp) | 50 | | | |
| Exposure time (ms) | 300 | | | |
| Reconstruction resolution (Voxel Size, Slice Thickness, Isotropic voxel size (µm)) | 250 | | | |
| Binning | 1:4 | | | |

For quantification, VivoQuant (InviCRO) was used to draw regions of interest (ROIs) in relevant tissues, with a ROI drawn over the left ventricle of the heart to serve as blood pool surrogate. Percentage injected dose per gram of tissue (%ID/g) was calculated from the tissue-associated radioactivity determined in the ROI, the volume of the ROI, and the decay-corrected dose entered during preprocessing of the images in VivoQuant.

**Efficacy and immune profiling studies**

***Calculation of tumor growth inhibition (TGI).*** Percent TGI is defined as the difference between the mean tumor volume of the test group and the control group. TGI values are calculated as follows:

| TGIᵢ [%] = | (1 − | Tᵢ − T_0_ | ) × 100 |
| --- | --- | --- | --- |
|  |  | Vᵢ − V_0_ |  |

T*ᵢ* is the mean tumor volume of a treatment group on a given day *i*, T₀ is the mean tumor volume of the treatment group on the day treatment started (day 0), V*ᵢ* is the mean tumor volume of the vehicle control group on the same day as T*ᵢ*, and V₀ is the mean tumor volume of the vehicle group on the initial day of treatment (day 0).

***Calculation of median survival time.*** The median survival time (MST) is the time at which the fractional survival equals 50%. MST was calculated using the GraphPad Prism software (Prism 8.4).

***Calculation of body weight loss.*** Relative body weight (RBW) was calculated as follows:

| RBW*ᵢ* [%] = | ( | BW*ᵢ* | −1 ) ×100 |
| --- | --- | --- | --- |
|  |  | BW_0_ |  |

BW*ᵢ* is the mean body weight of one group on a given day *i*, BW_0_ is the mean body weight of the same group on the day treatment started (day 0).

***Statistical analysis.*** For the evaluation of the statistical significance of antitumor efficacy, body weight change, and immune profiling, two-way ANOVA with multiple comparisons was performed using GraphPad Prism (Version 8.4).

The log-rank (Mantel–Cox) test for survival curve comparison was used (GraphPad Prism 8.4). All *P* values <0.05 were considered statistically significant.

Global significance statistic. Global significance statistic (GSS) is calculated from the t-statistics of gene set genes, which are calculated from linear regressions run in the DE analysis. GSS are calculated separately for each variable in the regression. For each covariate, it is calculated as the square root of the pathway’s average squared t-statistic, where t_i_ is the t-statistic from the i^th^ pathway gene:

| GSS = | ( | 1 | Σ | t^2^_i_ | ) | 1/2 |
| --- | --- | --- | --- | --- | --- | --- |
|  |  | *P* |  |  |  |  |
|  |  |  | *i*=1 |  |  |  |

**Irradiation and cell viability**

600 MCA205-mFAP cells per well in 384-well plates were seeded on day 0 and irradiated on day 1 using X-RAD 320 (Precision X-ray) with increasing amounts of radiation, and cell viability was measured on day 5 using Cell TiterGlo (Promega) on the Victor X (Perkin Elmer).

***In vitro* STING pathway activation analysis**

Cells were seeded at 1×10^6^ cells per well in 6-well plates the day before transfection with siRNA of cGAS, STING, TBK1, and IRF3 in duplicates, and with Non-Targeting Control (NTC, Dharmcon) by Lipofectamine RNAiMAX (Thermo Fisher Scientific) according to the manufacturer’s instructions. Cells were exposed to 10 Gy of radiation using an X-Rad320 (Precision X-ray) the following day; cells and supernatants were harvest 48 hours later. Total RNA from cells were isolated using PureLink mini kit (Thermo Fisher Scientific), cDNA was generated using qScript cDNA SuperMix, and qPCR was performed using TaqMan probe sets and master mix on the ViiA 7 system (Thermo Fisher Scientific). Chemokines were measured in supernatants using ELISA kits (R&D Systems) and detected on the Victor X (Perkin Elmer).
