# Supplementary material for: Fibroblast activation protein targeted radiotherapy induces an immunogenic tumor microenvironment and enhances the efficacy of PD-1 immune checkpoint inhibition
Source: Eur J Nucl Med Mol Imaging. 2023 Apr 22;50(9):2621–35. doi: 10.1007/s00259-023-06211-6 (PMC10317891; doi:10.1007/s00259-023-06211-6)
Supplement: Supplementary file 2 — Supplementary file2 (PDF 1035 KB) [file 259_2023_6211_MOESM2_ESM.pdf]

A

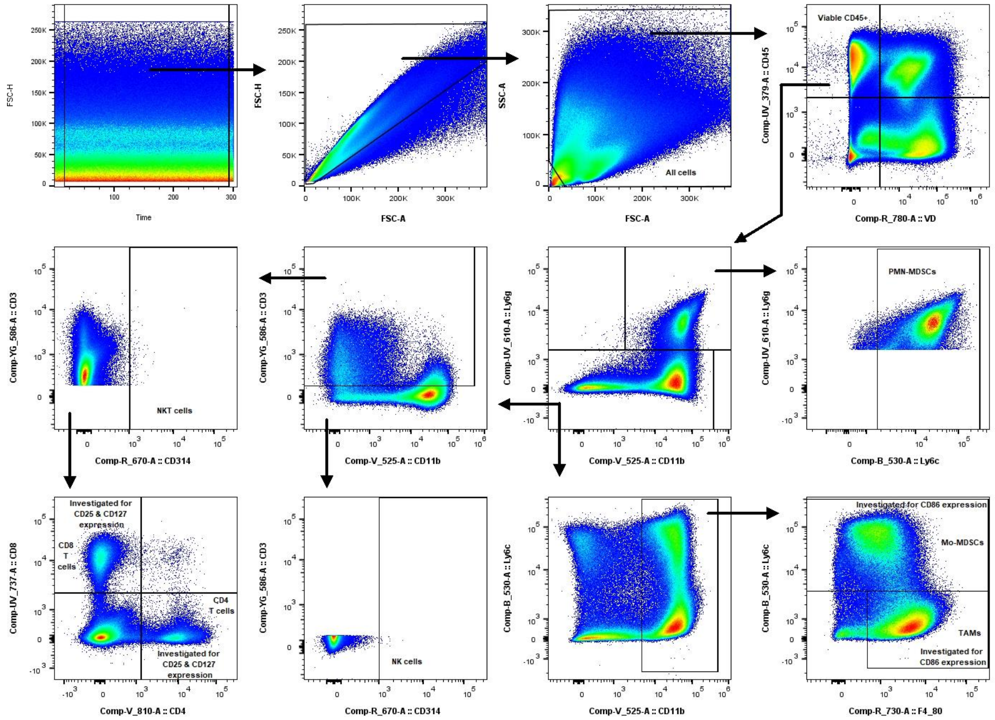

B

Cells	Markers
Immune cells	CD45+
PMN-MDSC	CD45+ CD11b+ Ly6c+ Ly6g+
Mo-MDSC	CD45+ CD11b+ Ly6c^{High} Ly6g-
TAM	CD45+ CD11b+ Ly6g- F4/80+ Ly6c-
T cells	CD45+ CD11b- Ly6g- CD3+ CD314-
CD8+ T cells	CD45+ CD11b- Ly6g- CD3+ CD314- CD8+
CD4+ T cells	CD45+ CD11b- Ly6g- CD3+ CD314- CD4+

Supplementary Figure S1. Gating strategy for immune profiling of MCA205-mFAP tumors by flow cytometry. Representative bivariate density plots show gating strategy to identify the PMN-MDSC, Mo-MDSC, TAM and T-cells in the MCA205-mFAP tumors (A). Marker sets used to identify each immune cell type are listed (B).

A

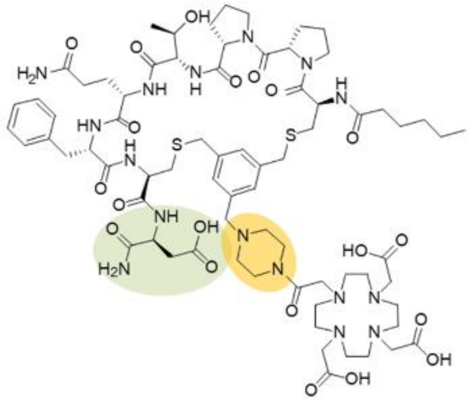

B

Binding of FAP-2287 and ^{nat}Lu-FAP-2287 to Human and Mouse FAP by SPR

Compound	***Human FAP K_D (nM, mean ± SD)***	***Mouse FAP K_D (nM, mean ± SD)***
FAP-2287	0.4 ± 0.1	1.2 ± 0.2
^{nat}Lu-FAP-2287	0.1 ± 0.0	0.5 ± 0.1

Inhibition of Human and Mouse FAP Endopeptidase Activity Assay

Compound	***Human FAP IC_{50} (nM, mean ± SD)***	***Mouse FAP IC_{50} (nM, mean ± SD)***
FAP-2287	1.4 ± 0.3	5.1 ± 0.5
^{nat}Lu-FAP-2287	1.3 ± 0.3	3.3 ± 0.4

Inhibition of Human DPP4 and PREP Endopeptidase Activity Assay

Compound	***Human DPP4 IC_{50} (nM, mean)***	***Human PREP IC_{50} (nM, mean)***
FAP-2287	>10000	>1000
^{nat}Lu-FAP-2287	>10000	>1000

Binding of FAP-2287 and ^{nat}Lu-FAP-2287 to Human FAP in a Cell Based Assay

Compound	***Human FAP IC_{50} (nM, mean ± SD)***
FAP-2287	3.9 ± 1.0
^{nat}Lu-FAP-2287	2.3 ± 0.3

Plasma Stability (24 h)

Compound	***Human Plasma (%)***	***Mouse Plasma (%)***
FAP-2287	>80	>80
^{nat}Lu-FAP-2287	>80	>80

K_D, equilibrium dissociation constant; IC_{50}, half-maximal inhibitory concentration; SD, standard deviation; SPR, surface plasmon resonance

Supplementary Figure S2. FAP-2287 structure (A) and *in vitro* characterization of FAP-2287 and ^{nat}Lu-FAP-2287 summary table (B).

A

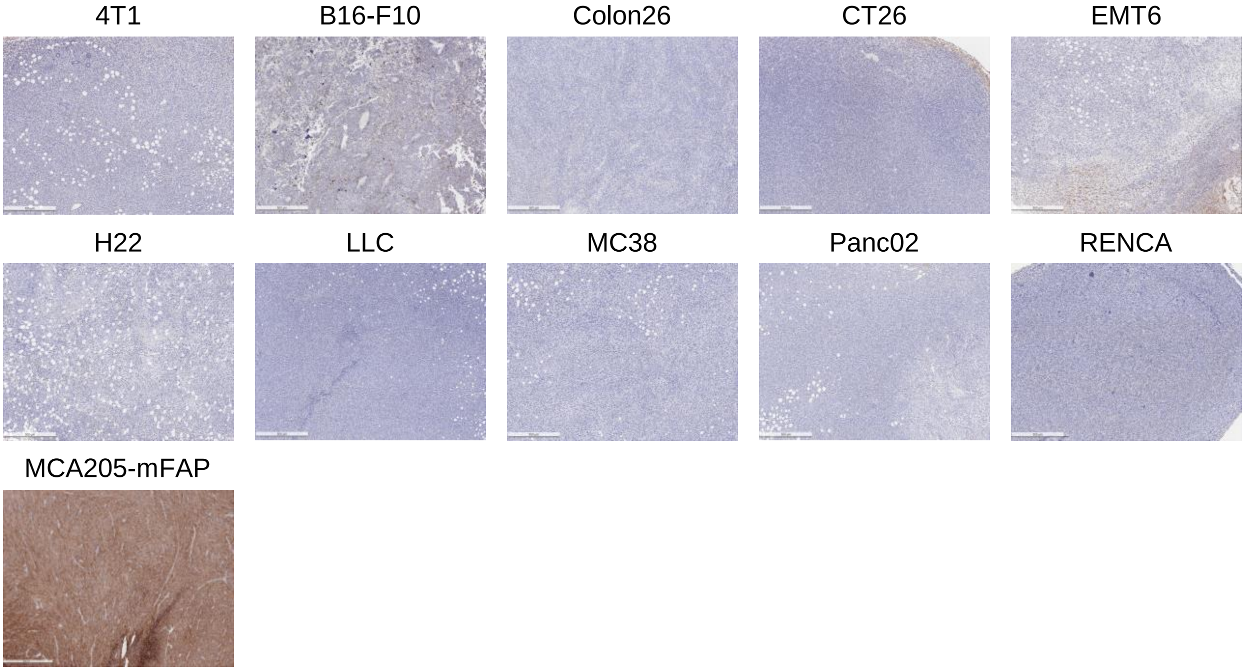

B

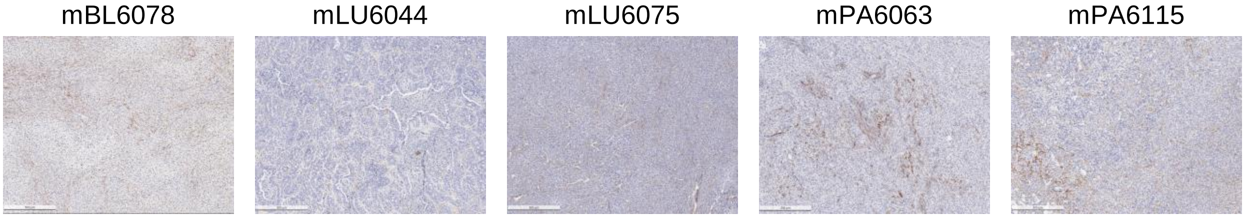

C

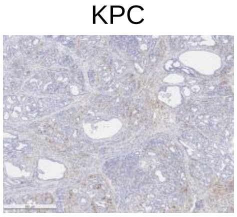

D

FAP Immunohistochemistry

Name	***Model type***	***Cancer type***	***H-score***
4T1	Syngeneic	Breast	8
B16-F10	Syngeneic	Melanoma	20
EMT6	Syngeneic	Breast	12
Colon26	Syngeneic	Colon	3
CT26.WT	Syngeneic	Colon	3
EMT6	Syngeneic	Breast	12
H22	Syngeneic	Liver	4
LLC	Syngeneic	Lung	4
MC38	Syngeneic	Colon	2
Panc02	Syngeneic	Pancreas	4
RENCA	Syngeneic	Kidney	4
MCA205-mFAP	Syngeneic	Fibrosarcoma	218
mBL6078	GEMM allograft	Bladder	28
mLU6044	GEMM allograft	Lung	13
mLU6075	GEMM allograft	Lung	15
mPA6063	GEMM allograft	Pancreas	8
mPA6115	GEMM allograft	Pancreas	32
KPC	GEMM	Pancreas	28

Supplementary Figure S3. FAP immunohistochemistry analysis of syngeneic, GEMM allograft, and GEMM tumors. Representative images show FAP staining in syngeneic (A, 500 μm), GEMM allograft (B, 500 μm), and GEMM tumors (C, 500 μm). Summary table of tumors with FAP quantification is shown (D).

A

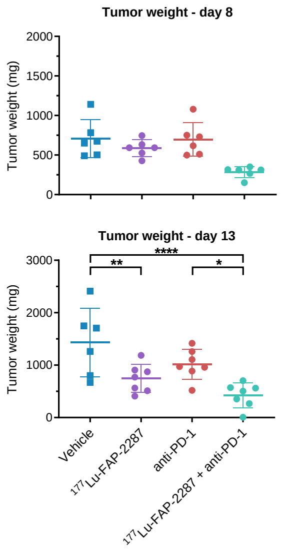

B

		Treatments			
Parameter	***Time point***	***Vehicle***	***^{177}Lu-FAP-2287***	***anti-PD-1***	***^{177}Lu-FAP-2287 + anti-PD-1***
Tumor weight (mg)	Day 8	706.8 ± 97.7	586.2 ± 43.4	698.2 ± 87.6	284.7 ± 28.8
	Day 13	1432.8 ± 267.1	746.0 ± 102.2**	1016.6 ± 108.6	423.9 ± 88.7****

Supplementary Figure S4. Tumor weight after treatment with ^{177}Lu-FAP-2287 and in combination with anti-PD-1 in MCA205-mFAP syngeneic tumor model. In the pharmacodynamic study, individual tumor weights on days 8 and 13 pi with bars as mean ± SEM (A) with the summary table of the data (B) are shown. Significant changes compared to vehicle in the summary table and between groups in the graph are denoted as * p<0.05, ** p<0.01, *** p<0.001 and **** p<0.0001.

A

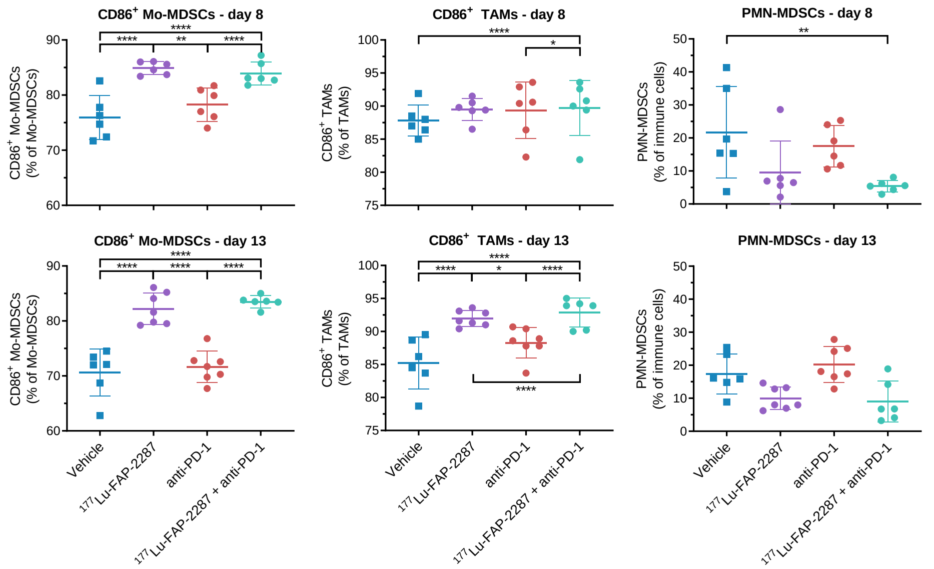

B

Immune population	Time point	Treatments: Vehicle	^{177}Lu-FAP-2287	anti-PD-1	^{177}Lu-FAP-2287 + anti-PD-1
CD86⁺ Mo-MDSCs (%, Mo-MDSCs)	Day 8	75.9 ± 4.0	84.9 ± 1.2****	78.3 ± 3.0	83.9 ± 2.1***
	Day 13	70.6 ± 4.3	82.2 ± 2.9****	71.7 ± 2.9	83.5 ± 1.1****
CD86⁺ TAMs (%, TAMs)	Day 8	87.8 ± 2.4	89.5 ± 1.7	89.4 ± 4.3	89.7 ± 4.1
	Day 13	85.2 ± 3.9	92.0 ± 1.2**	88.3 ± 2.3	92.9 ± 2.2**
PMN-MDSCs (%, CD45⁺)	Day 8	21.7 ± 5.7	9.6 ± 3.9	17.5 ± 2.6	5.4 ± 0.7**
	Day 13	17.4 ± 2.5	10.0 ± 1.3	20.3 ± 2.1	9.0 ± 2.5

C

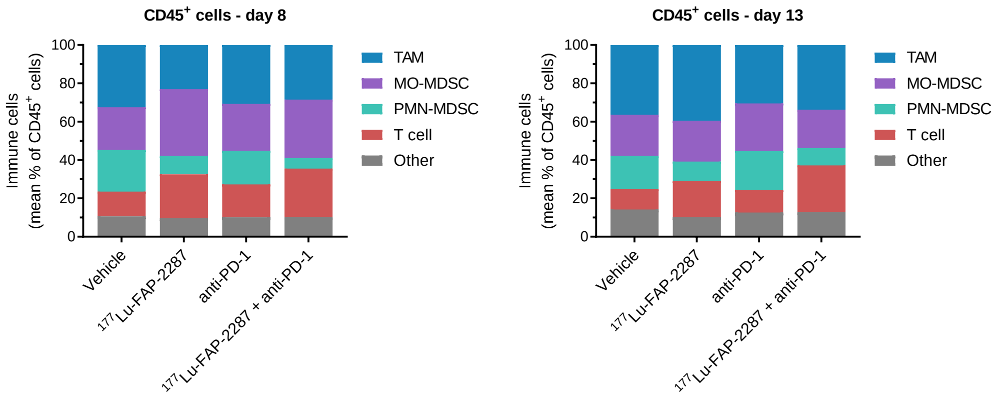

Supplementary Figure S5. Immune profiling of CD86⁺ Mo-MDSCs, CD86⁺ TAMs and PMN-MDSCs after treatment with ^{177}Lu-FAP-2287 and in combination with anti-PD-1 in MCA205-mFAP syngeneic tumor model. Individual percentage of CD86⁺ Mo-MDSCs, CD86⁺ TAMs and PMN-MDSCs with bars as mean ± SEM on day 8 and 13 pi (A), and a summary table (B) are shown. Mean percentages of lymphoid and myeloid subsets are plotted as stacked bars to denote changes relative to each population on day 8 (C, left) and 13 pi (C, right). Significant changes compared to vehicle in the summary table and between groups in the graph are denoted as * p<0.05, ** p<0.01, *** p<0.001 and **** p<0.0001.

A

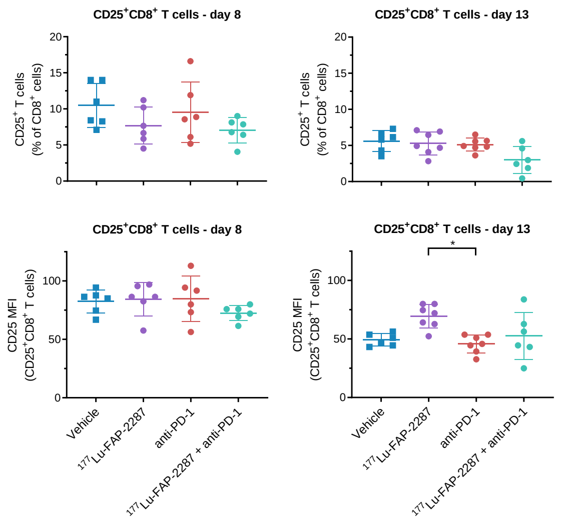

B

Immune population	*Time point*	*Vehicle*	*177Lu-FAP-2287*	*anti-PD-1*	*177Lu-FAP-2287 + anti-PD-1*
		Treatments			
$CD25^+CD8^+$ (%, $CD8^+$)	Day 8	10.5 ± 1.2	7.7 ± 1.1	9.5 ± 1.7	7.0 ± 0.7
	Day 13	5.6 ± 0.6	5.3 ± 0.6	5.1 ± 0.3	3.0 ± 0.8
CD25 on $CD25^+CD8^+$ (MFI)	Day 8	82 ± 4	84 ± 6	85 ± 8	72 ± 3
	Day 13	49 ± 2	70 ± 4	46 ± 3	53 ± 8

Supplementary Figure S6. Immune profiling of $CD25^+CD8^+$ T cells after treatment with ^{177}Lu-FAP-2287 and in combination with anti-PD-1 in MCA205-mFAP syngeneic tumor model. Individual percentage of $CD25^+CD8^+$ and their CD25 MFI with bars as mean ± SEM on day 8 and 13 pi (A), and a summary table (B) are shown. Significant changes compared to vehicle in the summary table and between groups in the graph are denoted as * p<0.05, ** p<0.01, *** p<0.001 and **** p<0.0001.

A

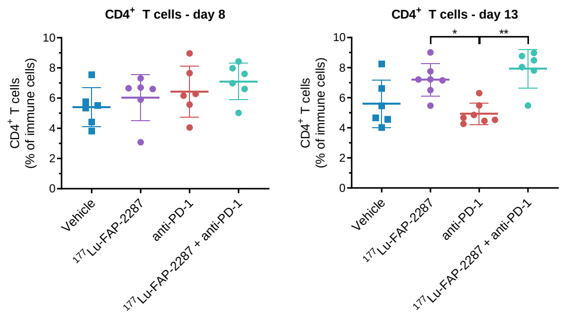

B

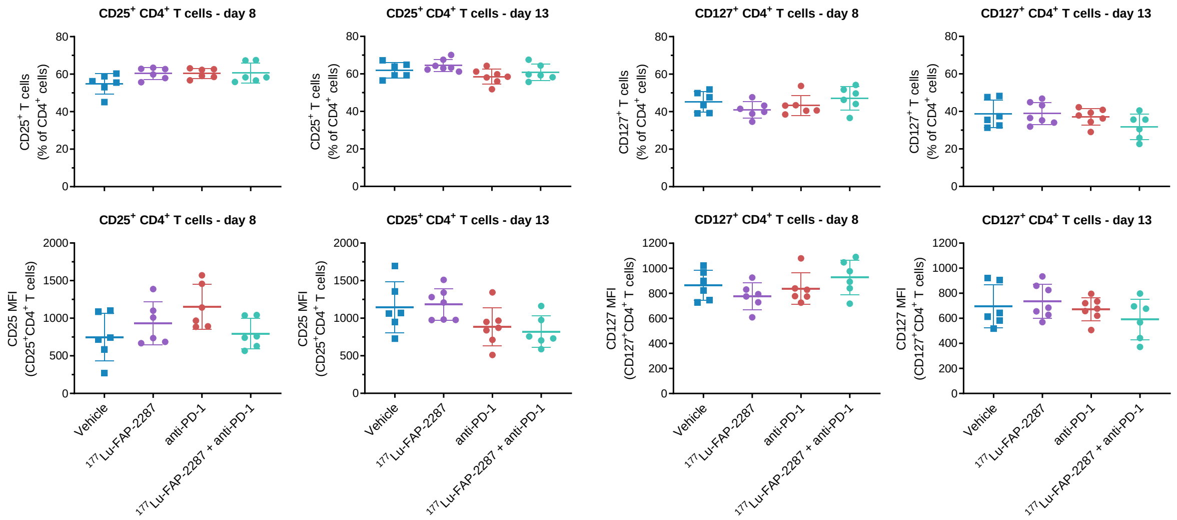

C

		Treatments			
Immune population	***Time point***	***Vehicle***	***177Lu-FAP-2287***	***anti-PD-1***	***177Lu-FAP-2287 + anti-PD-1***
$CD4^+$ (%, $CD45^+$)	Day 8	5.4 ± 0.5	6.0 ± 0.6	6.4 ± 0.7	7.1 ± 0.5
	Day 13	5.6 ± 0.6	7.2 ± 0.4	4.9 ± 0.3	7.9 ± 0.5
$CD25^+CD4^+$ (%, $CD4^+$)	Day 8	54.8 ± 2.2	60.4 ± 1.3	60.4 ± 1.1	60.6 ± 2.2
	Day 13	61.8 ± 1.7	64.5 ± 1.2	58.5 ± 1.5	60.8 ± 1.8
CD25 on $CD4^+$ (MFI)	Day 8	750 ± 128	931 ± 117	1153 ± 121	796 ± 83
	Day 13	1144 ± 138	1182 ± 80	885 ± 97	820 ± 86
$CD127^+CD4^+$ (%, $CD4^+$)	Day 8	45.2 ± 2.2	41.0 ± 1.8	43.3 ± 2.2	47.1 ± 2.6
	Day 13	38.8 ± 3.0	38.9 ± 2.2	37.1 ± 1.7	31.8 ± 2.7
CD127 on $CD4^+$ (MFI)	Day 8	864 ± 48	777 ± 43	837 ± 51	927 ± 57
	Day 13	697 ± 70	736 ± 51	673 ± 35	591 ± 66

Supplementary Figure S7. Immune profiling of $CD4^+$ T cells after treatment with ^{177}Lu-FAP-2287 and in combination with anti-PD-1 in MCA205-mFAP syngeneic tumor model. Individual percentage of $CD4^+$ T cells with bars as mean ± SEM on day 8 and 13 pi (A), individual percentage of $CD25^+CD4^+$ and $CD127^+CD4^+$ cells and their CD25 and CD127 MFI with bars as mean ± SEM on day 8 and 13 pi (B), and a summary table (C) are shown. Significant changes compared to vehicle in the summary table and between groups in the graph are denoted as * p<0.05, ** p<0.01, *** p<0.001 and **** p<0.0001.

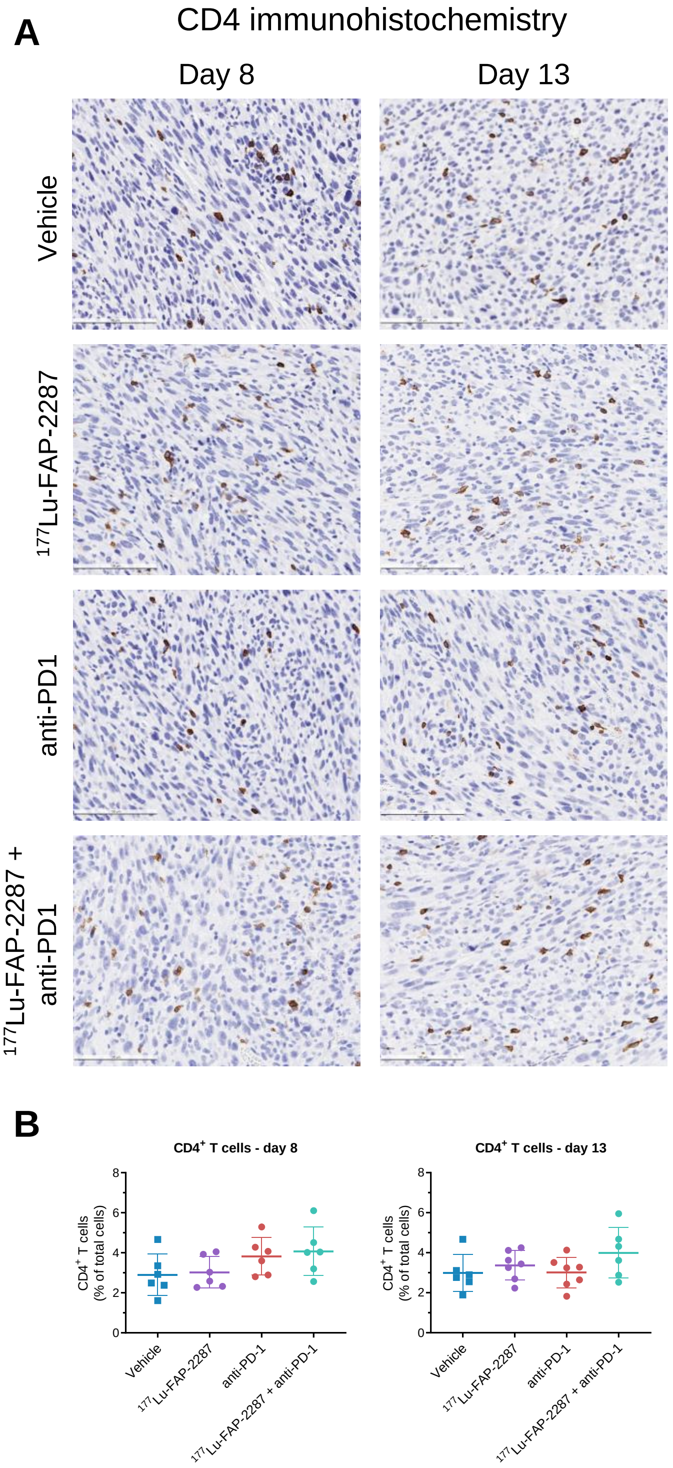

Supplementary Figure S8. IHC analysis of CD4+ T cells after treatment with ^{177}Lu-FAP-2287 and in combination with anti-PD-1 in MCA205-mFAP syngeneic tumor model. Representative images show CD4 staining in MCA205-mFAP tumors (A, 100 µm) and individual percentage of CD4+ T cells by IHC quantification with bars as mean ± SEM are plotted for days 8 and 13 pi (B). Significant changes between groups are denoted as * p<0.05, ** p<0.01, *** p<0.001 and **** p<0.0001.

A

Treatment and time point		*Treatment and time point*	*Shared genes*
^{177}Lu-FAP-2287 Day 8	vs	^{177}Lu-FAP-2287 + anti-PD1 Day 8	40
^{177}Lu-FAP-2287 Day 8	vs	^{177}Lu-FAP-2287 Day 13	16
^{177}Lu-FAP-2287 Day 13	vs	^{177}Lu-FAP-2287 + anti-PD1 Day 13	9
^{177}Lu-FAP-2287 + anti-PD1 Day 8	vs	^{177}Lu-FAP-2287 + anti-PD1 Day 13	27

B

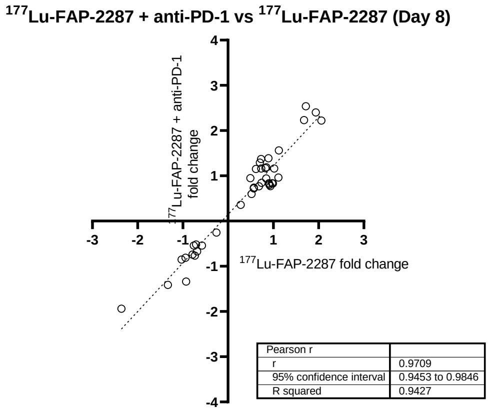

C

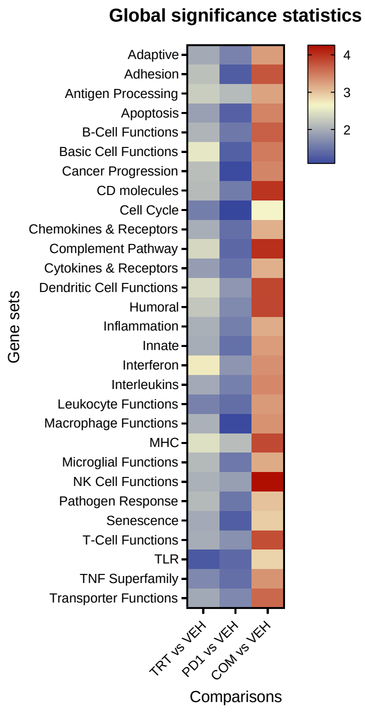

Supplementary Figure S9. Expression analysis of MCA205-mFAP tumors after treatment with ^{177}Lu-FAP-2287 and in combination with anti-PD-1. Comparison of genes that were significantly changed from vehicle control (P<0.05) show number of shared genes between ^{177}Lu-FAP-2287 and the combination with anti-PD-1 and between different timepoints (A). Comparison of fold changes to vehicle control for differentially expressed genes shared between single agent ^{177}Lu-FAP-2287 versus the combination with anti-PD-1 treatment show direct correlation with Pearson r of 0.9709 (B). Heatmap of global significance statistics for immune gene sets is shown for the different comparisons to vehicle control on day 8 pi (C). VEH, vehicle; TRT, ^{177}Lu-FAP-2287; PD1, anti-PD-1, COM, combination.

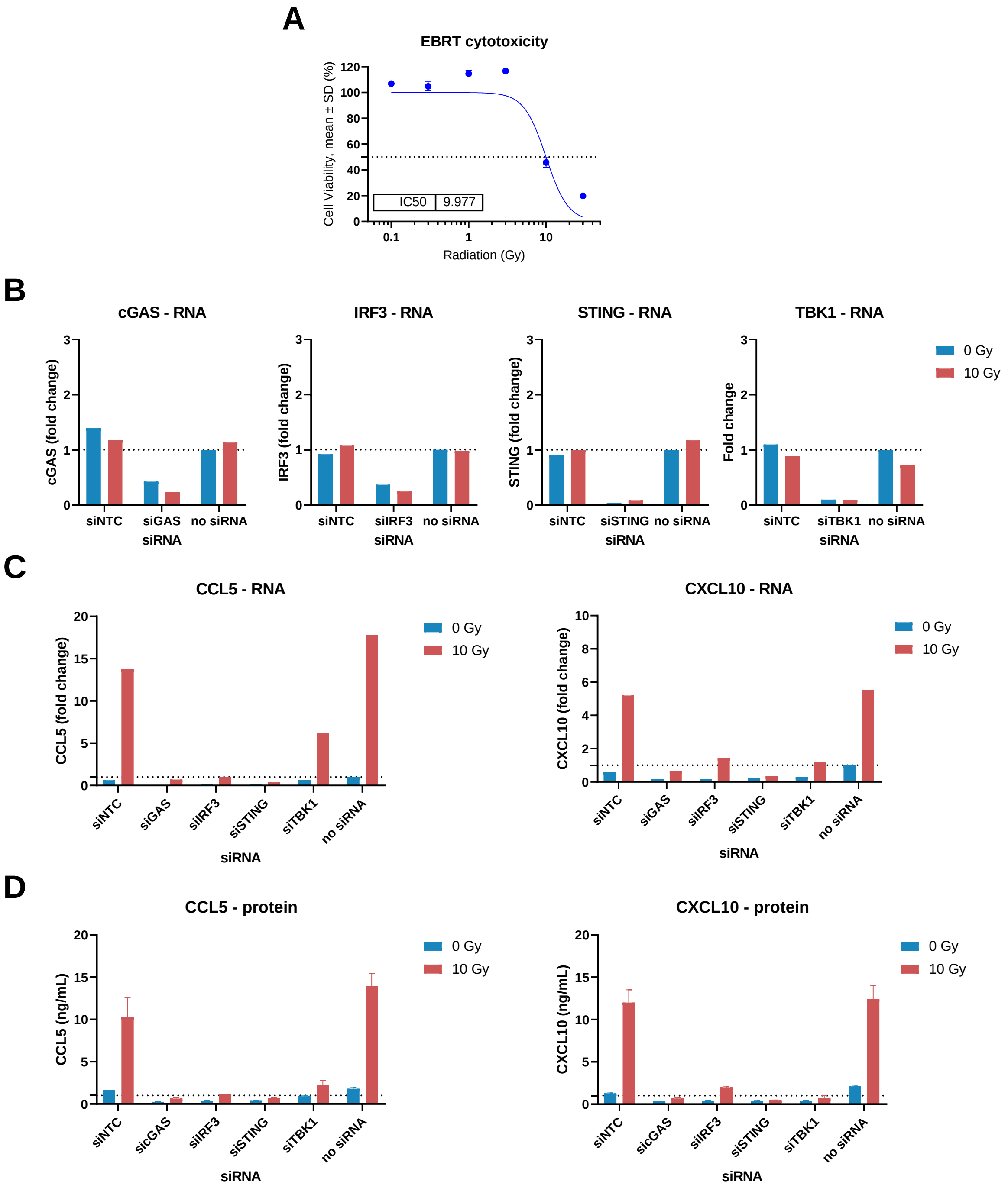

Supplementary Figure S10. Confirmation of STING pathway activation by radiation leads to upregulation of chemokines CCL5 and CXCL10 in MCA205-mFAP cells. The dose-response curve of MCA205-mFAP cells evaluated by cell viability at the indicated radiation doses. Data represent the mean ± SD performed in quadruplicate (A). Fold change in RNA expression of cGAS, IRF3, STING, and TBK1 after EBRT, in cells transfected with siRNA of the respective genes (B). Fold change in RNA expression of CCL5 and CXCL10 after EBRT, in cells transfected with siRNA of the STING pathway genes (C). CCL5 and CXCL10 cytokines levels in cultured supernatant, from cells transfected with siRNA of the STING pathway genes after EBRT (D).
